# Circular RNA expression profiles and *CircSnd1*-miR-135b/c-*foxl2* axis analysis in gonadal differentiation of protogynous hermaphroditic ricefield eel *Monopterus albus*

**DOI:** 10.1186/s12864-022-08783-3

**Published:** 2022-08-03

**Authors:** Zhi He, Zhijun Ma, Deying Yang, Qiqi Chen, Zhide He, Jiaxiang Hu, Faqiang Deng, Qian Zhang, Jiayang He, Lijuan Ye, Hongjun Chen, Liang He, Xiaoli Huang, Wei Luo, Shiyong Yang, Xiaobin Gu, Mingwang Zhang, Taiming Yan

**Affiliations:** 1 College of Animal Science and Technology, Sichuan Agricultural University, Chengdu, 611130 Sichuan China; 2 Luzhou City Department of Agricultural and Rural Affairs, Luzhou, 646000 Sichuan China; 3 Sichuan Water Conservancy Vocational College, Chengdu, 611231 Sichuan China; 4 College of Veterinary Medicine, Sichuan Agricultural University, Chengdu, 611130 Sichuan China

**Keywords:** circRNA, *Monopterus albus*, Foxl2, Gonadal differentiation, Reproductive, Expression pattern

## Abstract

**Background:**

The expression and biological functions of circular RNAs (circRNAs) in reproductive organs have been extensively reported. However, it is still unclear whether circRNAs are involved in sex change. To this end, RNA sequencing (RNA-seq) was performed in gonads at 5 sexual stages (ovary, early intersexual stage gonad, middle intersexual stage gonad, late intersexual stage gonad, and testis) of ricefield eel, and the expression profiles and potential functions of circRNAs were studied.

**Results:**

Seven hundred twenty-one circRNAs were identified, and the expression levels of 10 circRNAs were verified by quantitative real-time PCR (qRT–PCR) and found to be in accordance with the RNA-seq data, suggesting that the RNA-seq data were reliable. Then, the sequence length, category, sequence composition and the relationship between the parent genes of the circRNAs were explored. A total of 147 circRNAs were differentially expressed in the sex change process, and GO and KEGG analyses revealed that some differentially expressed (such as novel_circ_0000659, novel_circ_0004005 and novel_circ_0005865) circRNAs were closely involved in sex change. Furthermore, expression pattern analysis demonstrated that both *circSnd1* and *foxl2* were downregulated in the process of sex change, which was contrary to mal-miR-135b. Finally, dual-luciferase reporter assay and RNA immunoprecipitation showed that *circSnd1* and *foxl2* can combine with mal-miR-135b and mal-miR-135c. These data revealed that *circSnd1* regulates *foxl2* expression in the sex change of ricefield eel by acting as a sponge of mal-miR-135b/c.

**Conclusion:**

Our results are the first to demonstrate that circRNAs have potential effects on sex change in ricefield eel; and *circSnd1* could regulate *foxl2* expression in the sex change of ricefield eel by acting as a sponge of mal-miR-135b/c. These data will be useful for enhancing our understanding of sequential hermaphroditism and sex change in ricefield eel or other teleosts.

**Supplementary Information:**

The online version contains supplementary material available at 10.1186/s12864-022-08783-3.

## Background

Circular RNAs (circRNAs) are a class of endogenous noncoding RNAs characterized by covalently closed continuous loops, single strands and no free 3' or 5' terminus [1, 2]. In the past few years, the use of high-throughput sequencing technologies has demonstrated the widespread existence of circRNAs in nematodes [3, 4], zebrafish [5], fruitflies [6], mice [4, 7], pigs [8] and humans [4, 9]. Although the function of most circRNAs is largely unclear, current studies have revealed that some circRNAs are important modulators that regulate gene expression at multiple levels [4, 10–17].

CircRNAs have important biological functions during the development of the reproductive system [18–22]. Expression pattern analysis of many circRNAs in germ cells has shown typical developmental stages or tissue-cell specific characteristics, and their biological functions may be related to follicular development, ovarian senescence and spermatogenesis [23–26]. For example, *circINHA* has been shown to promote pig granulosa cell proliferation and inhibit granulosa cell apoptosis via *CTGF* as a competing endogenous RNA (ceRNA) that directly binds miR-10a-5p [27]. During spermatogenesis in mice, a large number of circRNAs with open reading frames are synthesized and then translated into proteins to compensate for the shortage of proteins caused by uncoupling of transcription and translation [28]. In medaka (*Oryzias latipes*), *circ880* may be related to gonadal development by combining with miR-375-3p [22]. Despite these sporadic reports, the functional role and molecular mechanism of circRNAs in the reproductive system is still in its infancy.

*Foxl2*, a female sex-associated gene, is principally expressed in granulosa cells and important for ovarian development and maintenance [29–35]. In mice, primary follicles are not formed and cannot complete maturation when *foxl2* function is lost, which may be caused by granulosa cells being reprogrammed into testis-specific Sertoli-like cells in a cell autonomous and oocyte-independent manner [35, 36]. Mechanistically, *foxl2* induces XX male sex reversal mainly by activating testis-specific genes (notably *Dmrt1* and *Sox9*) and repressing ovary-specific genes (notably *Cyp19*) [35, 37–39]. Research in olive flounder indicated that the expression of *foxl2* may be repressed via maintenance of DNA methylation [40]. In goats, *foxl2* mRNA has been detected in testes at several developmental stages, but no FOXL2 protein has been detected, suggesting that *foxl2* undergoes translational or posttranscriptional regulation [41]. Lines of evidence have shown that the expression of *foxl2* is significantly downregulated by miRNAs in humans and mice [42–45]. All of these results suggested that *foxl2* undergoes transcriptional or posttranscriptional regulation.

Ricefield eel is a protogynous hermaphroditic fish that changes from female to male through the intersex stage during its lifetime. Ricefield eel has been gradually recognized as a new model species for the study of biological development and evolution due to its sex change and small genome [46–48]. The expression level of *foxl2* in the ovary of ricefield eel has been shown to be high before sex change but to decrease sharply after the gonad developed into the ovotestis and testis [49–51]. Downregulation of *foxl2* has been thought to be responsible for gonadal differentiation of ricefield eel, but what and how *foxl2* expression declines is still unclear [51]. As important modulators involved in the transcriptional and posttranscriptional regulation of gene expression and miRNA sponges, circRNAs may be involved in the decreased expression of *foxl2* in the sex change of ricefield eel. However, there are no reports about circRNAs in ricefield eel to date. In the present study, we first performed transcriptome analysis in ricefield eel gonads to identify the potential circRNAs involved in ricefield eel sex change. Then, bioinformatics was used to analyse the function of differentially expressed circRNAs and their interaction with miRNAs. Finally, a dual luciferase reporter assay and RNA immunoprecipitation (RIP) were used to demonstrate the ceRNA relationship between *circSnd1*, *foxl2*, mal-miR-135b and mal-miR-135c. To our knowledge, this study is the first to analyse the expression profile of circRNAs in the gonads of fish with sex change, providing insights into the identification of novel targets for sex change in ricefield eel.

## Results

### Preliminary analysis of circRNA sequencing

Fifteen circRNA libraries from the gonads of ricefield eel at different developmental stages were constructed and sequenced, and the overview data for each library are shown in Additional file 1: Table S1. Briefly, the most and least raw and clean reads were obtained from IM1 (IM: middle intersexual gonad) and OV3 (OV: ovary), respectively. Clean base data of all libraries were > 15Gb, with Q20 > 97%, Q30 > 94%, error rate < 0.04%, GC content between 46% and 48%, and the ratio of clean reads mapped to the unique genomes was 76.46%-85.26%.

**Fig. 1:**
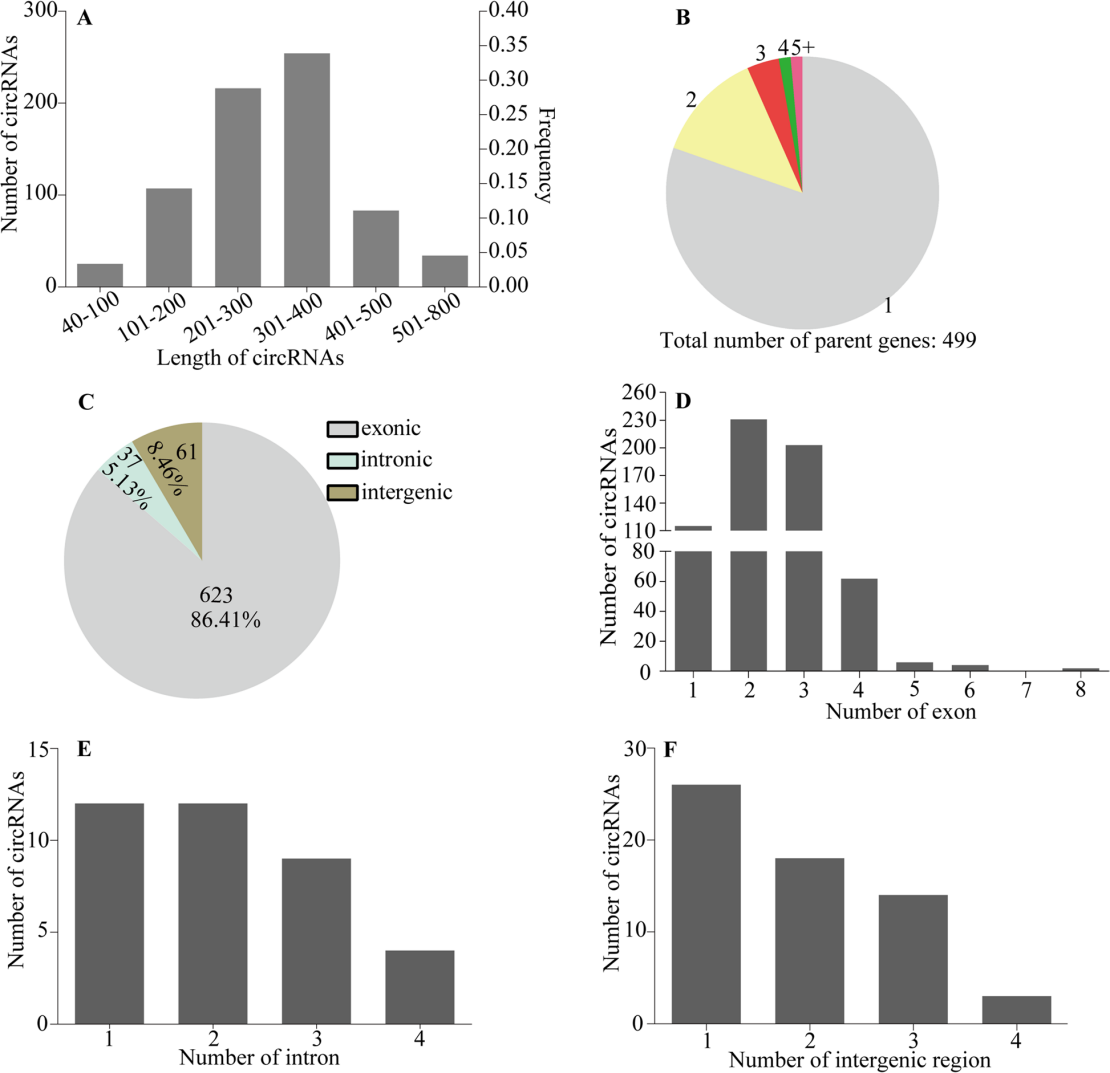
Characterization and classification of circRNAs. **A** CircRNA length and the number of circRNAs in the corresponding length range. **B** The relationship between the paternal genes and the circRNAs. “1” means that one paternal gene corresponds to one circRNA, “2” means that one paternal gene corresponds to two circRNAs, and so on. **C** CircRNA category and the number and proportion of circRNAs in the corresponding category. One circRNA contains one or more exons (**D**), introns (**E**), and intergene regions (**F**)

After rigorous selection, 721 circRNAs were identified from the 15 libraries, and all the circRNAs were novel because this was the first study to identify circRNAs in ricefield eel. The sequences and junction sequences of all the circRNAs are listed in Additional file 2: Data Base. Size distribution analysis revealed that the length of the 719 circRNAs ranged from 40-800 nt, the average length was 307 nt, and the majority (65.37%) ranged from 201-400 nt (Fig. 1A). Specifically, the lengths of novel_circ_0002103 (1619 nt) and novel_circ_0004589 (1480 nt) were significantly longer than those of the others. Statistical analysis of the parental genes showed that the 721 circRNAs were generated from 499 precursor mRNAs by back-splicing, and 80.36% of the precursor mRNAs produced one circRNA (Fig. 1B). Further statistics showed that all the circRNAs were exonic, intronic and intergenic, of which the majority were exonic (Fig. 1C). The numbers of exons, introns and intragenic regions contained in the circRNAs are also shown in Fig. 1D-F.

### Analysis of differentially expressed circRNAs

**Fig. 2:**
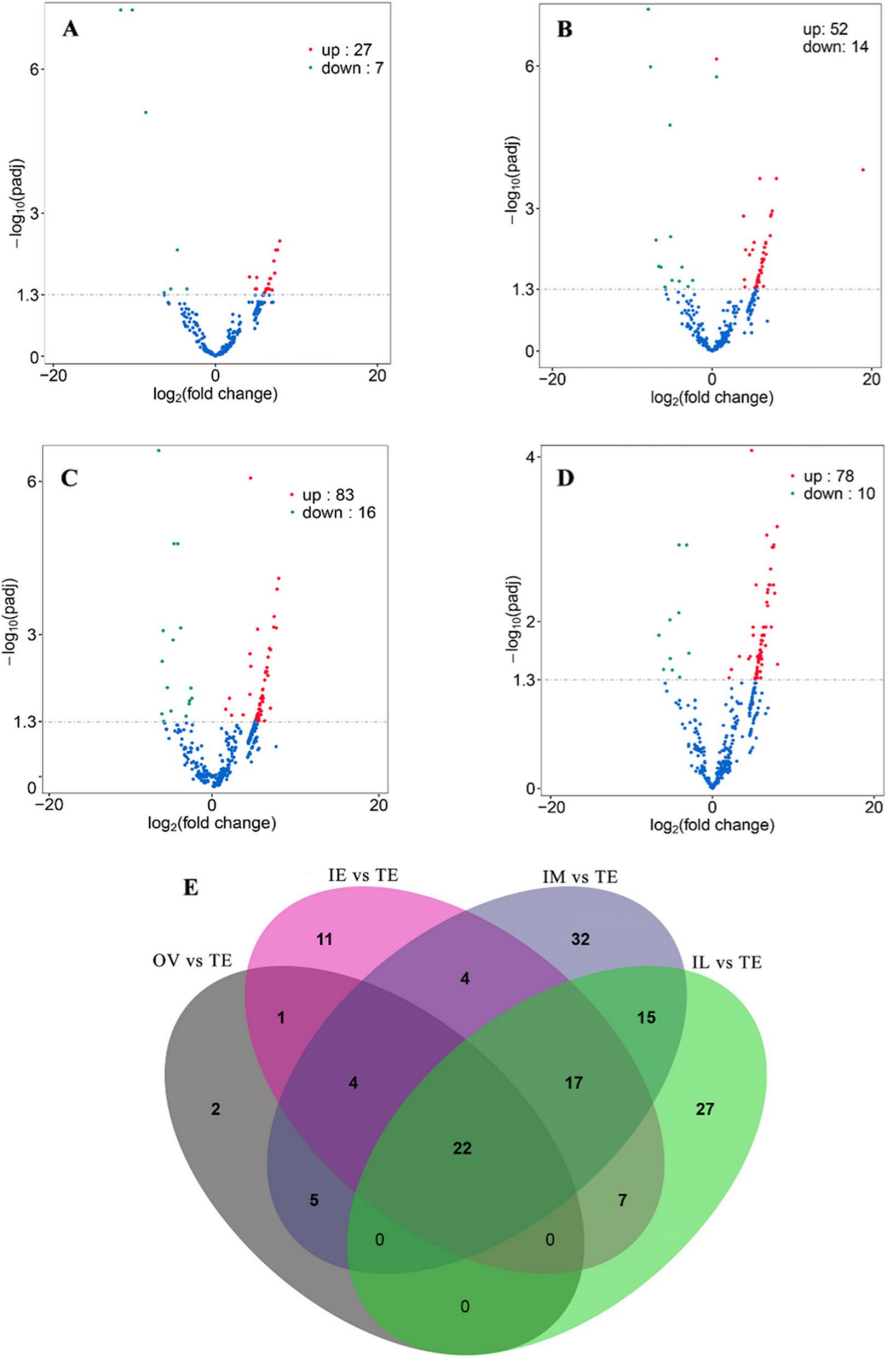
Differentially expressed circRNAs during gonadal development. **A** OV vs. TE. **B** IE vs. TE. **C** IM vs. TE. **D** IL vs. TE. **E** Venn diagram of differentially expressed circRNAs. OV: ovary, IE: early intersexual gonad, IM: middle intersexual gonad, IL: late intersexual gonad, TE: testis

The expression patterns of 147 differentially expressed (DE) circRNAs during the development of gonads are shown in the heatmap (Additional file 3: Fig. S1). In all, 82.31% of the DE circRNAs were strongly downregulated in the testis. Compared with the TE (testis), the number of DE circRNAs in the OV was the lowest (34 DE circRNAs), gradually increased, reached a peak (99 DE circRNAs) in IM vs. TE, and then gradually decreased (Fig. 2A-D). Notably, the expression of 22 circRNAs differed significantly during the whole sex change of ricefield eel (Fig. 2E and Additional file 4: Table S2).

### Functional analyses of the parent genes of the DE circRNAs

**Fig. 3:**
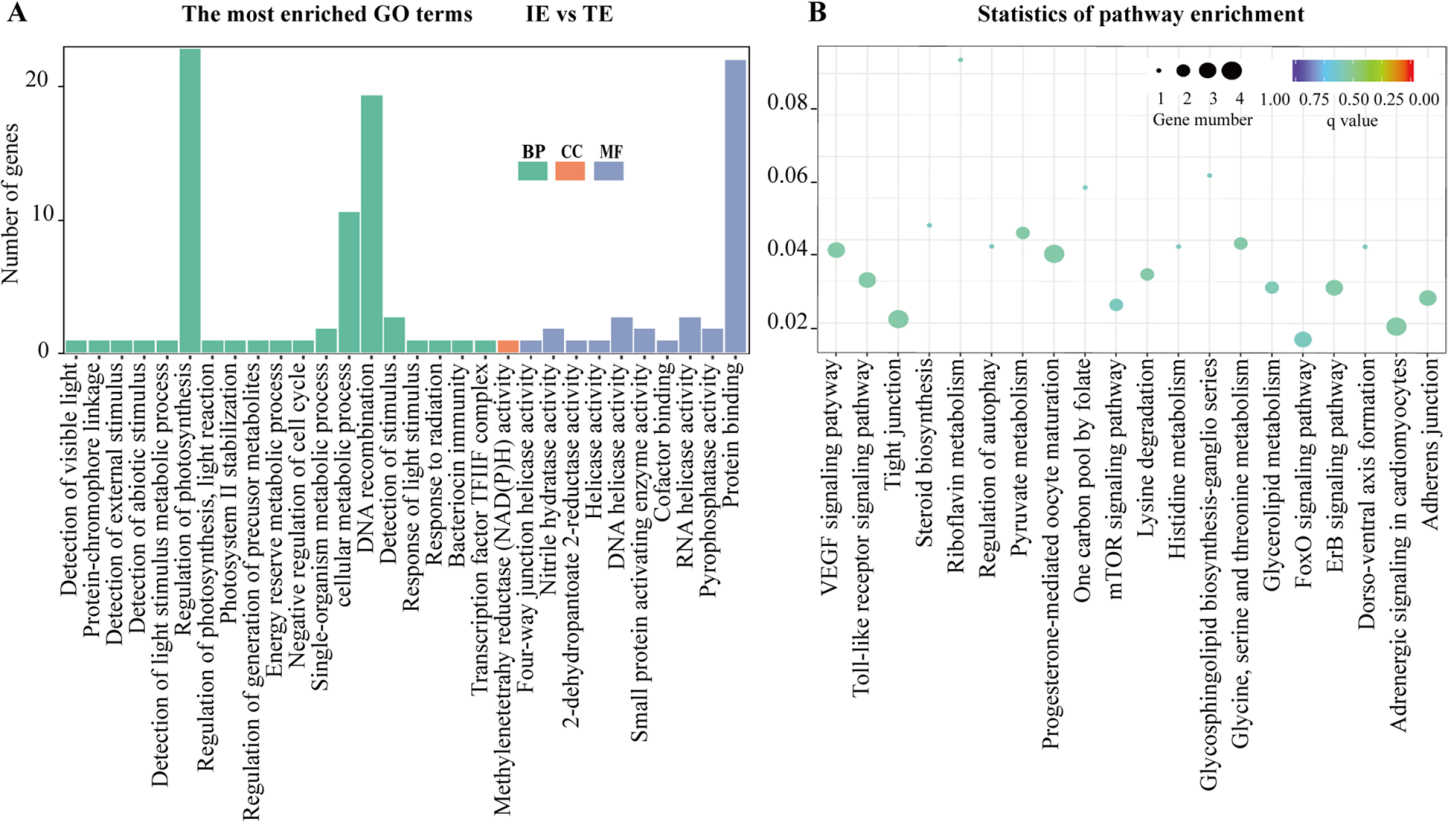
GO and KEGG enrichment of the parent genes of the DE circRNAs. **A** GO enrichment of the parent genes of the DE circRNAs in the IE vs. TE groups. IE: early intersexual gonad, TE: testis, BP: biological process, CC: cellular component, MF: molecular function. **B** KEGG enrichment of the parent genes of the DE circRNAs

The function of circRNAs could be reflected to some extent by their parent genes. Gene ontology (GO) term analysis showed that the parent genes extensively participated in many biological processes (Fig. 3A and Additional file 5: Fig. S2A-C). Biological process terms were abundant among the 4 groups, but cellular component terms were rarely involved and even absent in the OV vs. TE group (Additional file 5: Fig. S2A). Furthermore, the number of main functional groups of the parent genes increased gradually in the process of sex change. In addition, notably, the GO term “regulation of photosynthesis” was enriched in each group, but the number of genes enriched in this GO term was the largest in the IE vs. TE groups (Fig. 3A). PDZ domain and LIM domain 2 (*pdlim2*), the parent gene of novel_circ_0000659, was enriched in this term.

KEGG enrichment analysis was performed for all the parent genes of DE circRNAs. The results indicated that the top 20 most highly enriched (*P*-adj<0.05) pathways were involved in reproduction, such as the Toll-like receptor signalling pathway, progesterone-mediated oocyte maturation, mTOR signalling pathway and FoxO signalling pathway (Fig. 3B). More detailed analysis showed that 10 parent genes of 13 DE circRNAs were involved in many pathways (Additional file 6: Table S3). For example, *pik3r3*, *LOC109952263*, *aldh2* and *akt1* were involved in 13, 9, 13 and 14 pathways, respectively. Notably, *pik3r3* and *akt1* seem to be sequentially differentially expressed during the sex change of ricefield eel, *pik3r3* in OV/IM vs. TE, and *akt1* only in IL vs. TE. In addition, *spire2* and *pc* were enriched in all 4 groups, but *gab1*, *aldh2* and *akt1* were enriched in only one group. A comparative analysis of circRNAs in Additional file 4: Table S2 and Additional file 6: Table S3 shows that only novel_circ_0005856, novel_circ_0003251 and novel_circ_0003252 are common.

### Target miRNAs of the DE circRNAs

**Fig. 4:**
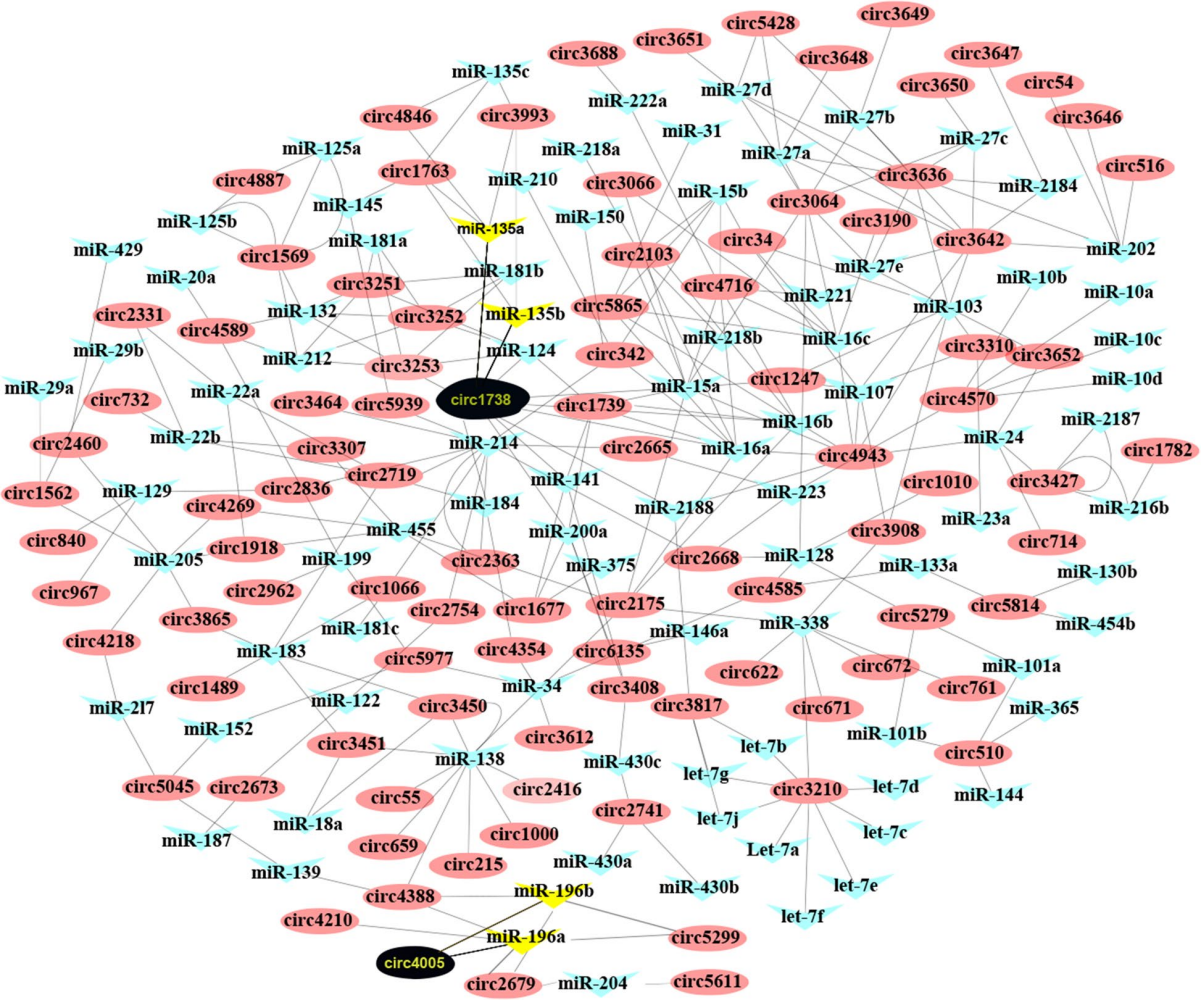
Schematic diagram of interactions between DE circRNAs, miRNAs and mRNAs. For presentation purposes, “novel_circ_000” and  "mal-miR-" were shortened to “circ” and "miR-", respectively in the diagram

The results showed that 96 DE circRNAs interacted with 91 miRNAs. Most of the DE circRNAs could act as miRNA sponges, and correspondingly, one miRNA could also be bound by several circRNAs. For example, novel_circ_0004943 can interact with 10 miRNAs, and mal-miR-16a can interact with 8 circRNAs (Fig. 4).

Novel_circ_0001738 (named *circSnd1*) and novel_circ_0004005 interact with mal-miR-135b/c and mal-miR-196a/b, respectively. In addition to mal-miR-135b/c, *circSnd1* also interacts with another 7 miRNAs (mal-miR-124, mal-miR-141, mal-miR-15a, mal-miR-16a/b, mal-miR-184 and mal-miR-200a). mal-miR-135b/c has been shown to be differentially expressed during sexual change of ricefield eel (Fig. 7B). To elucidate the mechanism of ceRNA, we further predicted target mRNAs for mal-miR-135b/c and mal-miR-196a/b. The results indicated that mal-miR-135b/c and mal-miR-196 may bind to the 3' UTRs of *foxl2* (Additional file 7: Fig. S3A) and *akt1*, respectively (Additional file 7: Fig. S3C).

### Confirmation of circRNAs by PCR and RT–qPCR

**Fig. 5:**
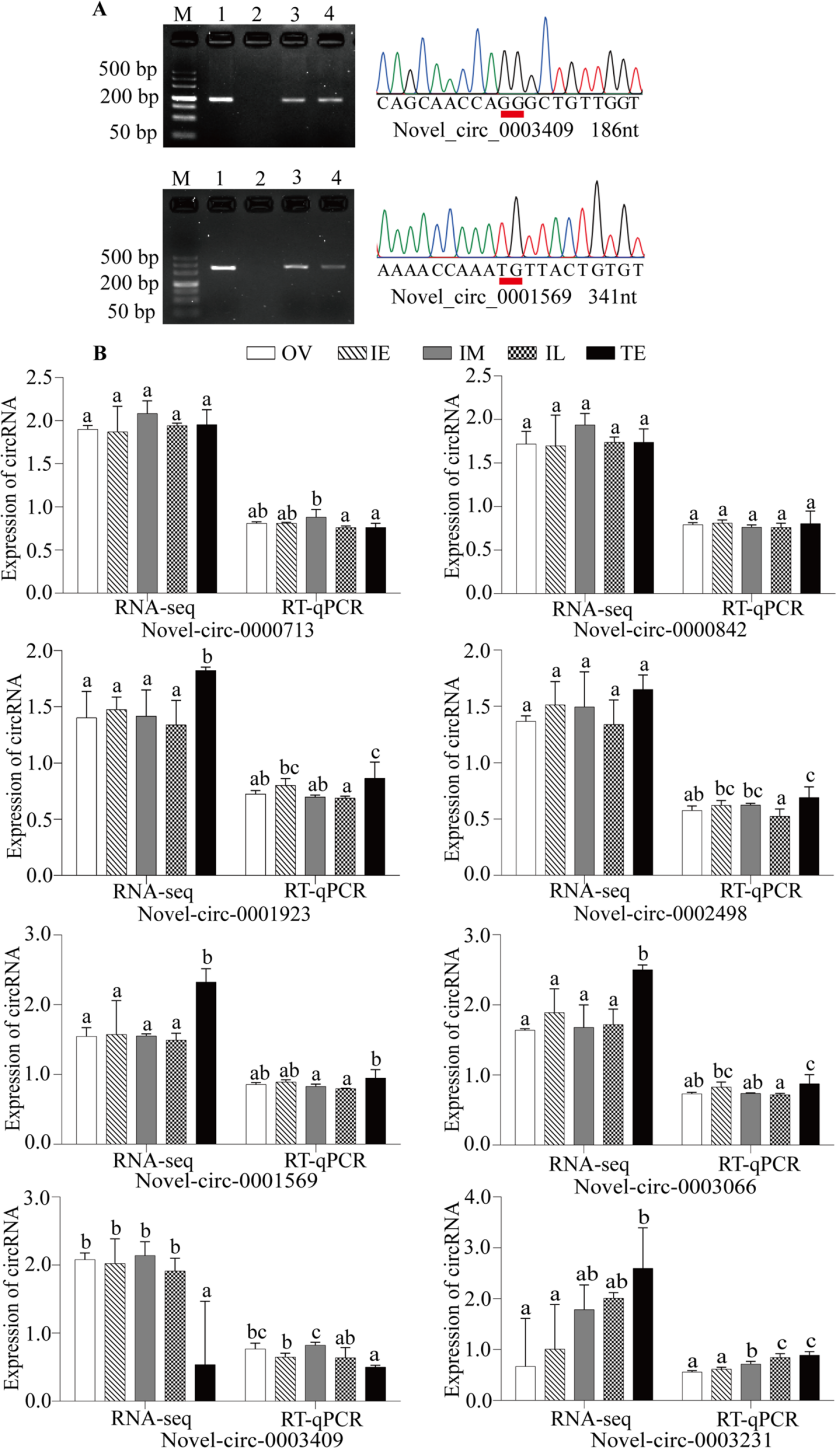
Characterization of the predicted circRNAs. **A** Amplification of the circRNAs using divergent primers with cDNA. Lanes 1, 3 and 4 refer to the cDNA samples transcribed by random hexamer primers, and lane 2 refers to the cDNA samples transcribed by oligo (dT)_18_. The cDNA used in lane 3 was synthesized by total RNA digested with RNase R, and lane 4 was a control.  : represents back-splicing sites. **B** Verification of circRNA expression patterns. The circRNA expression level of RNA-seq was calculated as Log_50_(TPM), and the RT–qPCR data were computed as the mean ± S.E.M. TPM: transcripts per million

We experimentally tested 10 candidate circRNAs from the gonads of ricefield eel by PCR and Sanger sequencing. Five DE and 5 non-differentially expressed (NDE) circRNAs were randomly chosen, and all 10 circRNAs were validated as expertly described. Specifically, circRNA forms were amplified in cDNA samples transcribed by random hexamer primers but not in cDNA samples transcribed by Oligo(dT)_18_ primers, and RNase R resistance experiments showed that circRNAs could protect against the degradation of RNase R (Fig. 5A and Additional file 8: Fig. S4). Moreover, Sanger sequencing further confirmed the circRNA sequencing and back-splicing sites.

To further verify the accuracy of the RNA-seq data, the aforementioned 10 circRNAs were quantified. The results indicated that the expression patterns of 8 out of 10 circRNAs were highly symmetrical with those of RNA-seq (Fig. 5B), and only novel_circ_0000873 and novel_circ-0001675 were not validated (data not shown). Although some subtle differences may exist, we consider them objective and acceptable. In summary, the RNA-seq results were reliable and precise.

### *CircSnd1* as a miRNA sponge of mal-miR-135b/c

Among the 4 DE circRNAs (novel_circ_0000659, *circSnd1*, novel_circ_0004005 and novel_circ_0005865) mentioned above, novel_circ_0004005 has the lowest expression level (data not shown). Based on the binding sites of miRNA and circRNA, only miR-135b/c could specifically bind to the splice site of *circSnd1*. Thus, *circSnd1* was selected for further analyses in order to ensure that we could effectively avoid the interference of mRNA.

**Fig. 6:**
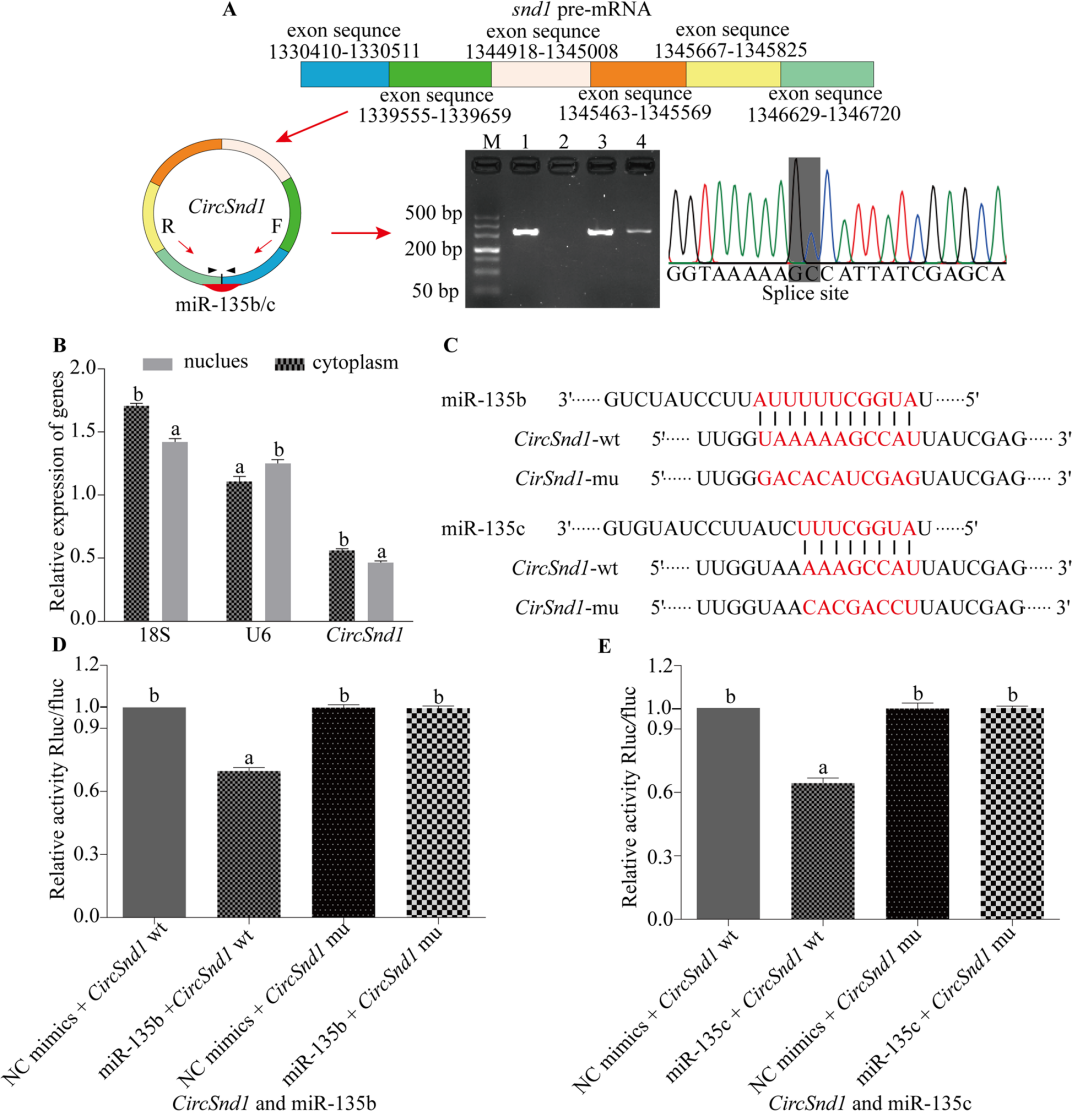
*circSnd1* interacts with mal-miR-135b/c. **A** The genomic loci of *circSnd1* and divergent primers were designed (F: forward primer, R: reverse primer), and the expected size of the product was amplified and verified by Sanger sequencing; the cDNA samples in lanes 1-4 were the same as those in Fig. 5A. **B** Quantitative results of *circSnd1* in the nucleus and cytoplasm. **C** Schematic representation of the mal-miR-135b/c target sequence within *circSnd1*. **D**, **E** Dual-luciferase assays for validating the interaction of mal-miR-135b/c with *circSnd1*. Values are the mean ± S.E.M., and the experiment was repeated at least three times. The letters above the bar chart indicated *P*<0.05

RNA-seq indicated that *circSnd1* was derived from 6 discontinuous exon sequences of *Snd1* pre-mRNA by back splicing, and bioinformatics analysis revealed that both mal-miR-135b and mal-miR-135c specifically bound to the back-splicing site of *circSnd1* (Fig. 6A). Cytoplasmic and nuclear separation of ricefield eel ovaries was performed to explore the location of *circSnd1*. U6 and 18S were demonstrated to be mainly expressed in the nucleus and cytoplasm, respectively, and were used as reference genes to test the expression level of isolated nucleoplasm RNA and confirm that *circSnd1* in the cytoplasm was detected (Fig. 6B), which further proved that *circSnd1* may act as a miRNA sponge of mal-miR-135b/c.

To further confirm this relationship, wt and mu pSI-*circSnd1* plasmids were constructed (Fig. 6C). As expected, there was a significant decrease in the luciferase activity mediated by wt-pSI-*circSnd1* when mal-miR-135b and mal-miR-135c mimics were cotransfected (Fig. 6D-E).

### *CircSnd1* was a potential modulator of *foxl2* by ceRNA

**Fig. 7:**
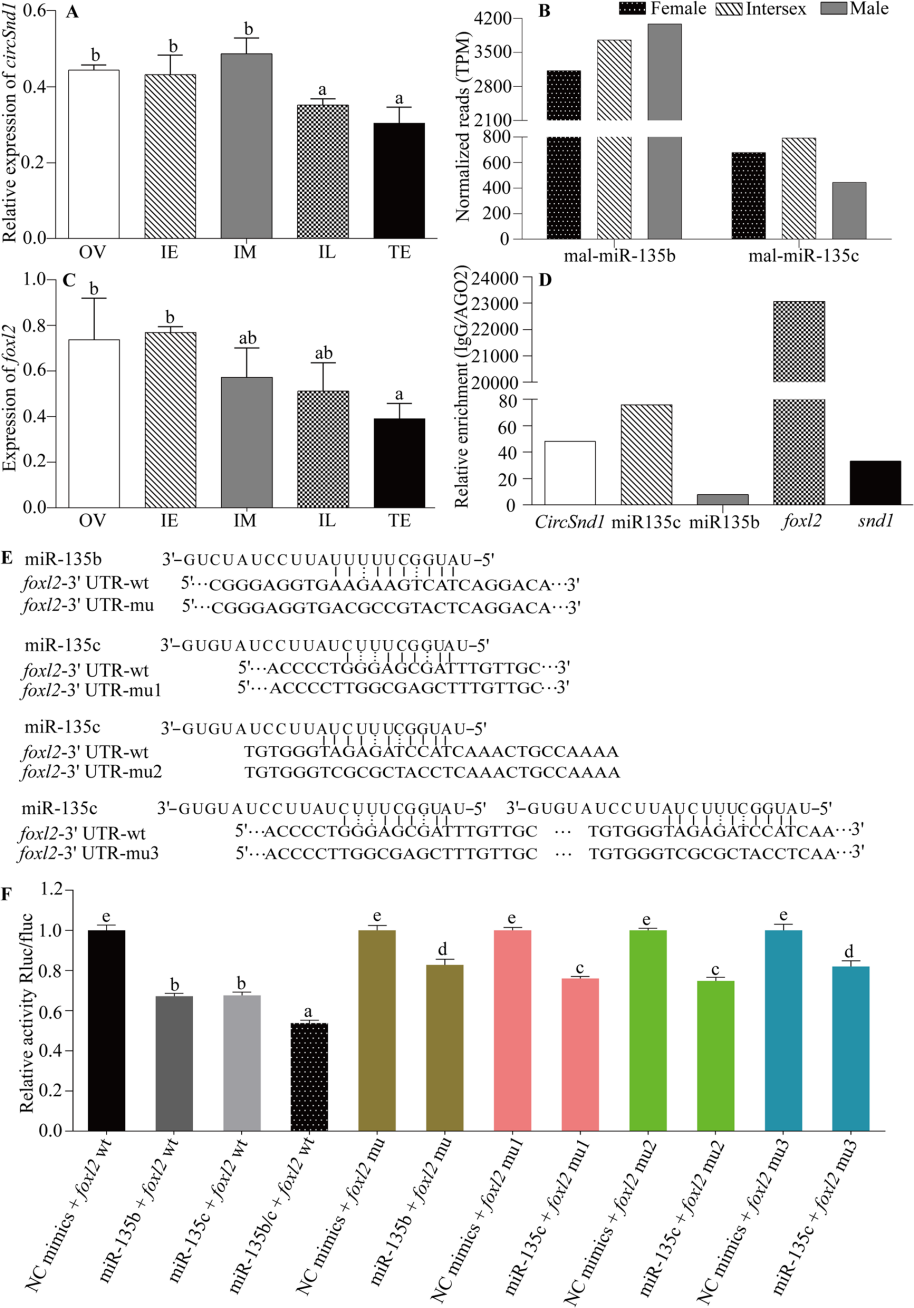
*CircSnd1* as a potential modulator of *foxl2*. **A**-**C** Expression patterns of *circSnd1*, mal-miR-135b/c and *foxl2* during the sex change. **D** *CircSnd1*, mal-miR-135b/c and *foxl2* were enriched in the RNA-induced silencing complex (RISC). **E** Schematic representation of the mal-miR-135b/c target sequence within the *foxl2* 3' UTR. **F** Dual-luciferase assays validating the -135b/c with *foxl2*. Values are the mean ± SEM, and the experiment was repeated at least three time. The letters above the bar chart indicated *P*<0.05

To further explore the potential biological function, the expression pattern of *circSnd1* during the sex change process was quantified. *circSnd1* was significantly downregulated in the IL and TE groups compared to the OV, IE and IM groups (Fig. 7A). Strongly increased expression of mal-miR-135b in the testes of ricefield eel compared to the ovaries and ovotestes (Fig. 7B). Obviously, the expression pattern of *circSnd1* was opposite that of mal-miR-135b, and *circSnd1* was a sponge of mal-miR-135b/c (Fig. 6). Based on these data, we speculated that there may be a target mRNA of mal-miR-135b/c in ricefield eel, whose expression is downregulated during the sex change process. *Foxl2* is a well-suited candidate target mRNA, because its expression is dramatically reduced in testes during the sex change (Fig. 7C). Alignment results of mal-miR-135b/c sequences with the *foxl2* 3' UTR also indicated that mal-miR-135b/c binding sites were detected in the *foxl2* 3' UTR (Additional file 7: Fig. S3A).

Based on the expression pattern, we performed an RIP test. As a component of the RISC enzyme in *Caenorhabditis elegans, Drosophila* and mammals [52], *snd1* was chosen as a control. The results revealed that although the miR135b enrichment multiple was low, all 5 RNAs were enriched in RISC (Fig. 7D). Remarkably, *foxl2* enrichment was abnormally higher than that of other genes. We then constructed wt and mu pSI-*foxl2* plasmids targeting binding sites (Fig. 7E), and a dual-luciferase reporter assay revealed that mutation of the mal-miR-135b/c binding sites completely abolished the inhibition of luciferase reporter activities (Fig. 7F). Taken together, our results strongly supported that mal-miR-135b/c binds to the *foxl2* 3' UTR.

## Discussion

### CircRNAs are important regulators of gene expression

CircRNAs express in multiple species that has the variety of the mechanism and molecular functions. Recent studies have detected 104388, 96675 and 82321 circRNAs in humans, macaques and mice, respectively [53]. A total of 70186 orthologous circRNAs exhibit conserved expression and splicing patterns across species, and numerous circRNAs may be involved in adaptive immunity, blood coagulation and neurotransmitters [53]. Current studies have shown that circRNAs can regulate gene expression extensively by circRNA-miRNA-mRNA regulatory network [54]. For example, the expression of miR-7 was inhibited by injecting zebrafish embryos with plasmid that can produce circular *CDR1as*, and then lead to significantly reduce of midbrain size [4]. *CircPIKfyve* acted as a ceRNA of miR-21-3p to reduce its inhibitory effect on *mavs* expression, which resulted in the activation of the NF-κB/IRF3 signaling pathway, and thereby enhancing the innate antiviral responses of Miiuy croaker [55]. *CircRasGEF1B* was also identified in Miiuy croaker with the same mechanism [56]. In addition to regulating gene expression, some circRNAs, such as *circ-ZNF609*, could be translated [28, 57]. Therefore, the research of the circRNAs function in sex determination and gonadal differentiation has great potential for understanding the intricate molecular events that underpin the extraordinary sex plasticity of fish.

CircRNAs were differentially expressed during sex reversal of ricefield eel. In other fish, 5052, 3762, 3868, 975 and 2727 circRNAs were identified in grass carp [58], half-smooth tongue sole [59], zebrafish [5], large yellow croaker [60] and medaka [22], respectively. In our study, the expression pattern of circRNAs in sex reversal of ricefield eel has been analyzed. A total of 721 circRNAs were obtained from the ovary, ovotestis and testis of protogynous hermaphroditic ricefield eel, and 144 DE circRNAs were identified from sex reversal. Then, 96 DE circRNAs interacted with 91 miRNAs. Bioinformatics analysis revealed that the DE circRNAs may play the important functions in the sex change process of ricefield eel, and the function of *circSnd1* was preliminarily revealed, which initiated the dawn of the study of circRNAs in fish gonads and sex change in fish. Evidence from mammals has demonstrated that circRNAs act as ceRNAs to regulate sex determination or gonadal differentiation cues [10, 12, 27, 61]. The above results suggest that DE circRNAs may play a key role in sex reversal of ricefield eel.

### DE circRNAs and their parent genes involved in regulation of sex change

GO and KEGG function enrichment of DE circRNAs were analyzed, the results revealed that some parent genes of circRNAs may be involved in sex change of ricefield eel. For example, the parent genes of novel_circ_0000659 (*pdlim2*), novel_circ_0004005 (*akt1*), and *spire2* (novel_circ_0005865).

The parent gene *pdlim2* may be a potential photosensitive gene in ricefield eel that closely involved in reproduction*.* In our study, *pdlim2* was enriched in the GO term of “regulation of photosynthesis”. Pdlim2 is characterized by a PDZ domain (protein–protein interaction domains) in the N-terminus and an LIM domain in the C-terminus, and played a very important biological role in organ development by actin anchorage [62]. *Plidm2* is strongly expressed in the corneal epithelium [63], high expression was detected in the inner and outer layers and lenses of zebrafish [64]. Opsins are the core of vision and are expressed primarily in ciliary photoreceptor cells, such as double cones and long and short single cones [65]. The consistent expression pattern of *pdlim2* and opsins led us to make the bold assumption that Pdlim2 has a function similar to that of opsins. As we known, light is one of the important environmental factors in the reproductive process for seasonally breeding animals [66]. Light can affect gonadal development and sex hormone secretion in fish. In California grunion (*Leuresthes tenuis*, *Ayres*), a higher proportion of females were found at long photoperiod, while short photoperiod treatment produced the most male-biased ratios [67]. Furthermore, the irradiation with light of a specific wavelength can trigger female-to-male sex reversal of medaka [68]. Pdlim2 is an insulin-like growth factor-I-regulated protein [69], a study in juvenile rainbow trout (*Oncorhynchus mykiss*) revealed that a long photoperiod could upregulate IGF-I levels in plasma [70]. Therefore, it is suggested that *pdlim2*, as a potential photosensitive molecule, may mediate a photoperiodic pathway and activate the sex change process of ricefield eel. Moreover, the function of novel_circ_0000659 in the photoperiodic signalling pathway deserves attention.

Given the important role of the PI3K-Akt signalling pathway in ovarian development [71], many DE genes were enriched in the PI3K-Akt signalling pathway during ricefield eel gonadal development from ovary to ovotestis [72], and a recent study also showed that the PI3K-Akt signalling pathway was regulated by miR-430s, resulting in the influence of steroidogenesis and sex differentiation of ricefield eel [73]. We speculated that the PI3K-Akt signalling pathway plays an irreplaceable role in the sex change process of ricefield eel. Bioinformatics analysis showed that mal_miR_196a/b targeted both novel_circ_0004005 and the 3' UTR of *akt1* (Fig. 4 and Additional file 7: Fig. S3B-C). Studies have demonstrated that circRNAs can alter the expression of their parent genes [13–15]. Taken together, our present study revealed that nove_circ_0004005 may act as a ceRNA to regulate the expression of *akt1*, in turn playing a potential regulatory role in the sex change process.

The parent gene *spire2* is one isoform of two SPIRE protein genes (*spire1* and *spire2*) that play a key role in reproduction. SPIRE is one member of WH2-containing actin nucleators [74, 75]. The highest expression levels of *spire1* and *spire2* were detected in mouse oocytes [76]. Then, the high expression of *spire1* and *spire2* was specifically detected in rat Sertoli and germ cells and mouse spermatocytes, respectively, but only minor expression levels of *spire1* were detected in mouse testes [77–79]. SPIRE proteins can initiate actin polymerization by binding actin monomers to four WH2 domains in the central part of the proteins [75]. In the mouse ovary, SPIRE1 and SPIRE2 can mediate asymmetric spindle positioning by assembling an action network that serves as a substrate for spindle movement and then drive polar body extrusion by promoting assembly of the cleavage furrow [76]. In rat testes, SPIRE1 is an important regulator of Sertoli cell actin and microtubule polymerization [79]. For example, *spire 1* knockdown leads to gross disruption of F-actin and microtubule organization across the seminiferous epithelium, which prevents spermatids from crossing the blood- testis barrier [79]. In the present study, only the parent gene *spire2* of novel_circ_0005865 was detected and enriched in all 4 groups. On this basis, we speculate that *spire2* may be an important molecule for sex changes in ricefield eel.

### *CircSnd1* regulates *foxl2* expression by functioning as a miR-135b/c sponge

*CircSnd1* and *foxl2* had similar expression patterns during the sex change of ricefield eel, and both of them could bind miR-135b/c through sequence complementary pairing. It was suggested that *circSnd1* may regulate *foxL2* expression through ceRNA mechanism to take part in the sex change of ricefield eel. Our present study demonstrated that *circSnd1* was downregulated in the sex change process of ricefield eel, which was consistent with the *foxl2* expression pattern but contrary to mal-miR-135b. Mechanistically, *circSnd1* may act as mal-miR-135b/c sponge to downregulate *foxl2* expression. *Foxl2* has been found within rather well-developed seminiferous tubules, but it has never been strongly co-expressed with *Sox9* in the same cell [80]. Another study showed that Sox9 immunoreactivity appears only after induction of *foxl2* deletion [37]. A similar spatiotemporal pattern was observed in the gonadal development of *Oryzias luzonensis* [81]. Forced expression of *foxl2* in XY transgenic mice induces seminiferous tubule disorganization and the development of ovotestis-like gonads, while downregulation of *foxl2* leads to female-to male sex reversal [82]. In gibel carp, *foxl2a-B* deficiency leads to remarkably increased expression of some testis differentiation-related genes (*dmrt1s* and *sox9bs*) in the ovary but decreased expression of some oocyte-derived factors (*cyp19a1as* and *gdf9s*), which result in ovary development arrest and sex reversal [83]. Similar results have been found in *Nile tilapia* [39], zebrafish [84] and olive flounder [40]. All of the above evidence shows that *foxl2* is mutually exclusive with male-specific genes and may induce XX male sex reversal by repressing testis-specific genes and activating ovary-specific genes.

The expression level of *foxl2* has been shown to be dramatically decreased once the ovaries develop into the ovotestis and testis [51], and the results of the present study were consistent with this finding. In ricefield eel, a study demonstrated that disruption of *foxl2* could significantly decrease the expression of *cyp19a1a* and the level of serum E2 but significantly increase the expression of *dmrt1* and the levels of serum T and 11-KT [50]. Some scholars strongly believe that *foxl2* is an important candidate gene responsible for sex change in ricefield eel [50, 51]. The expression of *foxl2* can be suppressed by mal-miR-430a/b/c binding to the 3' UTR of *foxl2* in ricefield eel [73]. In the present study, we demonstrated that mal-miR-135b/c binds to *cirSnd1* and *foxl2*. The sharp downregulation of *circSnd1* in the sex change process and upregulation of mal-miR-135b led to an increase in mal-miR-135b/c in the free state and binding to *foxl2*, ultimately resulting in the downregulation of *foxl2*. Here, we report for the first time that circRNAs regulate *foxl2* expression in ricefield eel, even in a sex change fish model, providing clues for further research on the role of circRNAs in sex determination or gonadal development.

## Conclusion

In conclusion, our present study identified 721 circRNAs in the gonads of ricefield eel for the first time, and 144 DE cricRNAs were revealed in the process of sex change of ricefield eel. GO and KEGG uncovered a lot of circRNAs, mRNAs and signalling pathways, which were closely involved in reproduction. *CircSnd1* acts as a sponge of mal-miR-135b/c to reduce *foxl2*, and we strongly believe that the endogenous downregulation of *foxl2* may be the main factor initiating the sex change of ricefield eel. Although our results enlightened the role of circRNAs in ricefield eel sex determination, more attention needs to be paid to identify more circRNAs related to gonadal development and finally clarify the sex change of ricefield eel, and even the teleost.

## Methods

### Experimental eels and sampling procedure

Ricefield eels (body weight = 65.20±4.59 g, body length = 41.26±7.13 cm) were purchased from a local market in Chengdu, Sichuan Province, China. The fish were temporarily maintained in the laboratory under a natural temperature and photoperiod for 24 h. The fish were randomly selected and anaesthetized with MS-222 (100 μg/mL, Syndel, Washington, USA) for 10 minutes. The gonads were collected after the ricefield eels were dissected and divided into three parts: one was fixed in Bouin’s solution for 24 h and then stored in 75% alcohol until used for determination the gonadal developmental stage. The remaining two fragments were immediately frozen in liquid nitrogen and then stored at -80°C until use; and one fragment was sent to Novogene (Novogene, Beijing, China) for RNA extraction for “library construction and RNA-seq”, and the other fragment was used for RNA extraction for “PCR amplification and Sanger sequencing”, “real-time quantitative PCR analysis”.

**Fig. 8:**
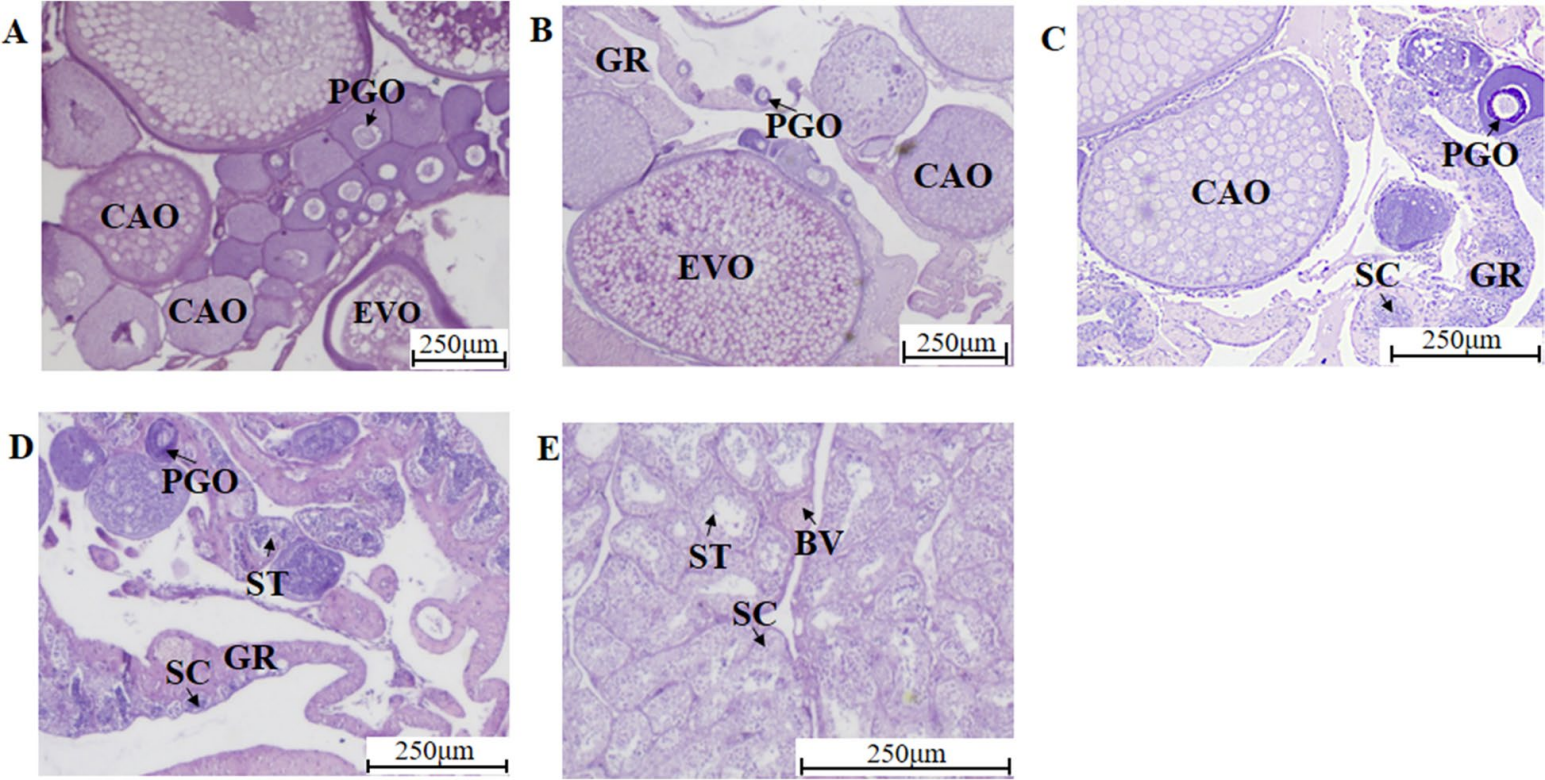
Results of identification of the gonad development stages of ricefield eel. **A** Ovary (OV), **B** early intersexual gonad (IE), **C** middle intersexual gonad (IM), **D** late intersexual gonad (IL), **E** testis (TE). CAO: cortical alveolar oocyte, PGO: primary growth stage oocyte, GR: gonadal ridge, SC: spermatocyte, ST: spermatid, BV: blood vessel, EVO: early vitellogenesis oocyte

Histological sections that were 5 μm thick and stained with haematoxylin-eosin were used to determine the developmental stage of ricefield eel gonads based on a previous study [85, 86]. In the present study, the ovary (OV), early intersexual gonad (IE), middle intersexual gonad (IM), late intersexual gonad (IL) and testis (TE) were used (*n* = 3, Fig. 8).

### Library construction and RNA-seq

Total RNA was extracted from the OV, IE, IM, IL and TE samples of ricefield eel using TRIzol reagent (Invitrogen, Carlsbad, CA, USA) according to the manufacturer’s instructions. RNA purity and contamination were assessed using a NanoPhotometer® spectrophotometer (IMPLEN, CA, USA), integrity was detected by 1% agarose gel electrophoresis, and concentration was measured using a Qubit® RNA Assay Kit in a Qubit® 2.0 Flurometer (Life Technologies, CA, USA). The RNA was qualified when A260/A280 was between 1.8 and 2.1, the brightness of the 28S band was approximately twice that of the 18S band, and there was no 5S band. The RNA samples of each stage were three biological replicates.

For circRNA enrichment, total RNA was treated with a Ribo-zero rRNA Removal Kit (Epicentre, Madison, USA) to remove ribosomal RNAs, and then 5 μg RNA was incubated with RNase R (Epicentre, USA) according to previously described procedures to remove linear RNAs [4]. Subsequently, the sequencing libraries were generated by the NEBNext® Ultra™ Directional RNA Library Prep Kit for Illumina® (NEB, USA) following the manufacturer’s recommendations. Briefly, divalent cations under elevated temperature in NEBNext First Strand Synthesis Reaction Buffer were used to fragment the RNA. First-strand cDNA was synthesized using random hexamer primers and M-MuLV Reverse Transcriptase (RNase H). Second-strand cDNA synthesis was subsequently performed using DNA Polymerase I and RNase H. In the reaction buffer, dNTPs with dTTP were replaced with dUTP. The remaining overhangs were converted into blunt ends via exonuclease/polymerase activities. After adenylation of the 3' ends of DNA fragments, NEBNext adaptors with hairpin loop structures were ligated to prepare for hybridization. To preferentially select cDNA fragments of 150~200 bp in length, the library fragments were purified with the AMPure XP system (Beckman Coulter, Beverly, USA). Then, 3 μl USER Enzyme (NEB, USA) was used with size-selected, adaptor-ligated cDNA at 37 °C for 15 min followed by 5 min at 95 °C before PCR. Then, PCR was performed with Phusion High-Fidelity DNA polymerase, universal PCR primers and Index (X) Primer. Finally, the products were purified (AMPure XP system), and library quality was assessed on an Agilent Bioanalyzer 2100 system. After cluster generation, the libraries were sequenced on an Illumina HiSeq 4000 platform, and 150 bp paired-end reads were generated. Library construction and sequencing were carried out by Novogene (Novogene, Beijing, China).

### circRNA identification

Raw data (raw reads) in FASTQ format were first processed through in-house prescripts. Clean reads were obtained by removing reads containing adapters, reads containing poly-N and low-quality reads from the raw data. At the same time, the Q20, Q30 and GC contents of the clean data were calculated. The reference genome (M_albus_1.0) was downloaded from NCBI (*Monopterus albus* - Nucleotide - NCBI) directly, and paired-end clean reads were aligned to the reference genome using Bowtie. Then, the circRNAs were detected and identified using find_circ [4] and CIRI_2_ [87].

### RNA isolation and cDNA synthesize

Total RNA was extracted from the gonads of ricefield eel using TRIzol reagent (Invitrogen, Carlsbad, CA, USA) according to the manufacturer’s instructions. gDNA was removed with gDNA Eraser (Trans Biotech, Beijing, China) before reverse transcription. The cDNAs were transcribed from the total RNA (1.0 μg) of OV, IE, IM, IL and TE using the RevertAid First Strand cDNA Synthesis Kit (Thermo Scientific, IN, USA, Cat#: K1622) according to the manufacturer’s instructions. Two kinds of cDNA were synthesized by random hexamer primers and oligo (dT)_18_ primers. In addition, cDNA was synthesized by random hexamer primers from the RNA, which was treated with RNase R (Epicentre, Biotechnologies Cat#: RNR07250) or ddH_2_O (control) as described in a previous study [4].

### PCR amplification and Sanger sequencing

To validate the circRNAs, 10 circRNAs were randomly selected for PCR confirmation. Primer 5 was used to design divergent primers (both side sequences of circRNA junctions) for each candidate circRNA (Additional file 9: Table S4), and the cDNA synthesized in the previous section of this section was used as template. In general, divergent primers were expected to amplify circRNAs from cDNA synthesized by random hexamer primers but could not amplify circRNAs from cDNA synthesized by Oligo (dT)_18_ primers.

PCR was performed in a 10 μL final volume containing 0.5 μL of each 10 μM primer solution, 5.0 μL of 2×Taq Master Mix (Vazyme, Nanjing, China), 3 μL of ddH_2_O and 1 μL of gonadal cDNA template. The PCR conditions were as follows: an initial 3 min denaturation at 95 °C; 36 amplification cycles of 0.5 min at 95 °C, 0.5 min at melting temperature (Tm, °C), and 1.0 min at 72 °C; followed by a final extension for 15 min at 72 °C. PCR products were detected by 3% agarose gel electrophoresis, target products from cDNA (by random hexamer primer) were ligated into T-Vector pMD^TM^19 (TaKaRa, Dalian, China) for T-A cloning, and Sanger sequencing was performed by TsingKE Biological Technology Company Limited (Chengdu, Sichuan, China).

### Real-time quantitative PCR analysis

The circRNAs selected above were further verified by quantitative real-time PCR (qRT–PCR) in gonads, and the primers were the same as those used to validate the circRNAs. Total RNA (100 ng) from gonad in OV, IE, IM, IL and TE stage (5 biological replicates for each stage) was reverse transcribed with random primers by using the RevertAid First-strand cDNA Synthesis Kit (Thermo Scientific, MA, USA) according to the manufacturer’s protocol, and the cDNA quality was verified by successful amplification of *rpl17*.

qRT–PCR was performed in a Bio–Rad CFX Connect system in a final reaction volume of 10 μl, comprising 5 μl of 2×SYBR Green Master Mix (TaKaRa, Dalian, China), 0.4 μL of each primer (10 μmol/l), 3.2 μL of nuclease-free water and 1 μL of cDNA template. The cycling parameters were 95 °C for 5 min, followed by 40 amplification cycles of 95 °C for 10 s, 59 °C for 15 s and 72 °C for 20 s. The specificity of qPCR amplification was confirmed by melting curve analysis, agarose gel electrophoresis, and sequencing of PCR products.

circRNA expression levels were quantified using a standard curve prepared with tenfold serial dilutions of the plasmid containing the corresponding DNA fragment, which ranged from 10^2^ to 10^9^ copies. The correlation coefficients and PCR efficiencies were not less than 0.990 and 90%, respectively. To minimize variation due to differences in cDNA loading, the expression levels of the target circRNAs were normalized to the geometric mean expression levels of *rpl17* and *ef1α*. Target gene expression was calculated by ${\mathsf{C}}_{\mathsf{target\ circRNA}}/\sqrt{{\mathsf{C}}_{\mathsf{ef1}{\alpha}}\times {\mathsf{C}}_{\mathsf{rpl17}}}$.

### Target miRNA and mRNA prediction and bioinformatic analysis

The potential miRNA binding sites of differentially expressed (DE) circRNAs were predicted by RNA22 v2 microRNA target detection, miRanda and BiBiServ2 RNAhybrid. The miRNAs of ricefield eel have not been included in miRBase, so 160 ricefield eel miRNAs that were used as libraries for target prediction. The data for mal-miR-135b/c in Fig. 7B were both cited from Gao Y et al [88], and the data for *foxl2* in Fig. 7C were cited from our previous experiments [89]. Some sex-biased genes from our previous study or validated by other biologists were used as candidate mRNAs to predict miRNA binding sites. The circRNA-miRNA interaction networks were constructed by Cytoscape 3.3.0. Gene Ontology (GO) and Kyoto Encyclopedia of Genes and Genomes (KEGG) enrichment [90] were performed on all host genes of DE circRNAs to determine the potential role of circRNAs.

### Dual-luciferase reporter assay

The interactions between *circSnd1/*miR-135b/c and *foxl2*/miR-135b/c were measured using the Promega Dual-Luciferase System (Promega, Madison, USA). The *circSnd1* sequences containing wild-type (wt) or mutant (mu, A-C and G-T mutagenesis for 4 nucleotides) miR-135b/c binding sites were synthesized, and the sequences of *Xho*I and *Not*I were synthesized upstream or downstream, respectively. For the generation of the luciferase reporter construct, the wt and mu sequences were ligated into the pSI-Check2 (cloud-seq biotech, Shanghai, China) vector, which was digested with XhoI and NotI (Invitrogen, USA) restriction enzymes. The recombinant plasmids were purified with the PureLink™ HiPure Plasmid Maxiprep Kit (Invitrogen, USA) following the manufacturer’s recommendations and then cotransfected with miR-135b/c mimics or control mimics using Lipofectamine 2000 (Invitrogen, USA) into 293T cells (Sangon Biotech, Shanghai, China). Dual-luciferase activities were tested with the Luciferase Assay Reagent (Promega, Madison, USA) 48 h later.

### RNA immunoprecipitation

RIP was performed by using the Magna RIP RNA-Binding Protein Immunoprecipitation Kit (Millipore) according to the manufacturer’s instructions. Briefly, the gonads of ricefield eel were dispersed into single cells with a homogenizer and homogenized using RIP lysis buffer plus RNase inhibitor (Thermo Scientific) and proteinase inhibitor cocktail (Roche, Switzerland). After a soft freeze–thawing, 100 μL mixtures were then incubated with antibody targeting AGO2 (Invitrogen, Cat#: MA5-23515) or isotype IgG (Invitrogen, Cat#: 10400C) overnight at 4 °C, and 10 μL mixtures were taken as the input group. After washing six times with RIP wash buffer, the RNA–protein complex-associated beads were dissolved in proteinase K (Invitrogen, Cat#: 25530031) buffer, and then RNA was isolated with salt precipitation according to the manufacturer’s instructions, followed by quantitative reverse transcription PCR (RT–PCR) analysis. Primers for RT–PCR are shown in Additional file 10: Table S5. The enrichment levels were calculated by using POWER (0.5, Ct_Ago2_ - Ct_IgG_).

### Separation of cytoplasm and nucleus

To determine the cytoplasmic and nuclear localization of *circSnd1*, the cytoplasmic and nuclear RNA of the fresh ovaries of ricefield eel were extracted according to the manufacturer’s recommendations for the RNA Purification Kit (Norgen Biotek, Canada) and then reversed transcribed with the RevertAid First Strand cDNA Synthesis Kit (Thermo Scientific, IN, USA, Cat#: K1622) according to the manufacturer’s instructions. Small nuclear U6 and 18S were used as internal controls, and 3 biological duplicates and 5 technical duplicates were performed for each gene. Primers for RT–PCR are shown in Additional file 11: Table S6. The relative expression levels were normalized to the geometric mean expression levels of *rpl17* and *ef1α* by ${\mathsf{C}}_{\mathsf{circSnd1}}/\sqrt{{\mathsf{C}}_{\mathsf{ef1}{{\alpha}}}\times {\mathsf{C}}_{\mathsf{rpl17}}}$.

### Statistical analysis

GraphPad Prism 7 (GraphPad Software Inc., La Jolla, CA) was used to analyse the experimental data. The expression RNA levels were determined using one-way analysis of variance (ANOVA) followed by Duncan’s multiple comparisons test using SPSS 21.0 software (SPSS, Inc., Chicago, IL, USA). All the values are expressed as the mean ± S.E.M, and *P* < 0.05 was considered statistically significant.

## Supplementary Information

**Additional file 1.**

**Additional file 2.**

**Additional file 3.**

**Additional file 4.**

**Additional file 5.**

**Additional file 6.**

**Additional file 7.**

**Additional file 8.**

**Additional file 9.**

**Additional file 10.**

**Additional file 11.**

## Data Availability

The datasets generated and analysed during the current study are available in the Sequence Read Archive (https://www.ncbi.nlm.nih.gov/sra/) of National Center for Biotechnology Information database with accession number SRR17761834 to SRR17761848. The link for reviewers is “https://dataview.ncbi.nlm.nih.gov/object/PRJNA798587?reviewer=crqsh87e724odhieculi1mild”.The datasets analysed during this study are included in this published article and its supplementary information files. Please contact Zhi He (zhihe@sicau.edu.cn) if someone wants to request the data from this study.

## Abbreviations

circRNA: Circular RNA
ceRNA: Competing endogenous RNAs
mal-miR: *Monopterus albus miRNA*
3' UTR: 3' untranslated region
5' UTR: 5' untranslated region
OV: Ovary stage
IE: Early intersexual stage
IM: Middle intersexual stage
IL: Late intersexual stage
TE: Testis stage
RIP: RNA immunoprecipitation
RNA-seq: RNA sequencing.
