# Supplementary material for: Circular RNA expression profiles and CircSnd1-miR-135b/c-foxl2 axis analysis in gonadal differentiation of protogynous hermaphroditic ricefield eel Monopterus albus
Source: BMC Genomics. 2022 Aug 3;23:552. doi: 10.1186/s12864-022-08783-3 (PMC9347082; doi:10.1186/s12864-022-08783-3)
Supplement: Supplementary file 1 — Additional file 1. [file 12864_2022_8783_MOESM1_ESM.docx]

**Table S1 Summary of circRNAs sequencing data**

| Sample | Duplicates | Raw reads | Clean reads | clean bases | Error rate | | Q20 | Q30 | GC content | Unique mapped | Unmapped |
| --- | --- | --- | --- | --- | --- | --- | --- | --- | --- | --- | --- |
| OV | OV1 | 125771310 | 120693216 | 18.1G | 0.03 | 97.74 | | 94.35 | 47.69 | 84.09 | 12.88 |
|  | OV2 | 116716772 | 112163932 | 16.82G | 0.04 | 97.87 | | 94.46 | 48.12 | 82.59 | 12.81 |
|  | OV3 | 107260808 | 101886556 | 15.28G | 0.01 | 97.51 | | 93.68 | 48.71 | 82.7 | 12.57 |
| IE | IE1 | 122208478 | 117499704 | 17.62G | 0.01 | 97.93 | | 94.62 | 47.75 | 82.42 | 12.89 |
|  | IE2 | 118497904 | 113779752 | 17.07G | 0.02 | 97.97 | | 94.7 | 47.78 | 83.72 | 12.81 |
|  | IE3 | 112587104 | 108126242 | 16.22G | 0.01 | 97.98 | | 94.71 | 47.86 | 85.26 | 11.38 |
| IM | IM1 | 133142504 | 127272988 | 19.09G | 0.03 | 98.06 | | 94.91 | 48.1 | 82.82 | 13.47 |
|  | IM2 | 117994066 | 112682204 | 16.9G | 0.01 | 97.85 | | 94.42 | 48.21 | 82.9 | 12.8 |
|  | IM3 | 128995190 | 124438390 | 18.67G | 0.02 | 97.76 | | 94.26 | 47.95 | 84.93 | 11.14 |
| IL | IL1 | 126654934 | 121287964 | 18.19G | 0.01 | 97.86 | | 94.49 | 48.3 | 81.01 | 14.64 |
|  | IL2 | 130816696 | 124128046 | 18.62G | 0.01 | 97.82 | | 94.43 | 47.94 | 81.96 | 14.27 |
|  | IL3 | 110418234 | 105558106 | 15.83G | 0.01 | 97.77 | | 94.3 | 48.7 | 78.74 | 13.86 |
| TE | TE1 | 119342536 | 115074368 | 17.26G | 0.03 | 97.91 | | 94.61 | 47.06 | 77.84 | 16.59 |
|  | TE2 | 110474786 | 106618306 | 15.99G | 0.04 | 97.91 | | 94.66 | 46.63 | 77.03 | 17.49 |
|  | TE3 | 114047316 | 109011776 | 16.35G | 0.02 | 97.54 | | 93.69 | 48.06 | 76.46 | 15.75 |

*Note:* OV: ovary, IE: early intersexual gonad, IM: middle intersexual gonad, IL: late intersexual gonad, TE: testis.
