# Supplementary material for: Circular RNA expression profiles and CircSnd1-miR-135b/c-foxl2 axis analysis in gonadal differentiation of protogynous hermaphroditic ricefield eel Monopterus albus
Source: BMC Genomics. 2022 Aug 3;23:552. doi: 10.1186/s12864-022-08783-3 (PMC9347082; doi:10.1186/s12864-022-08783-3)
Supplement: Supplementary file 3 — Additional file 3. [file 12864_2022_8783_MOESM3_ESM.docx]

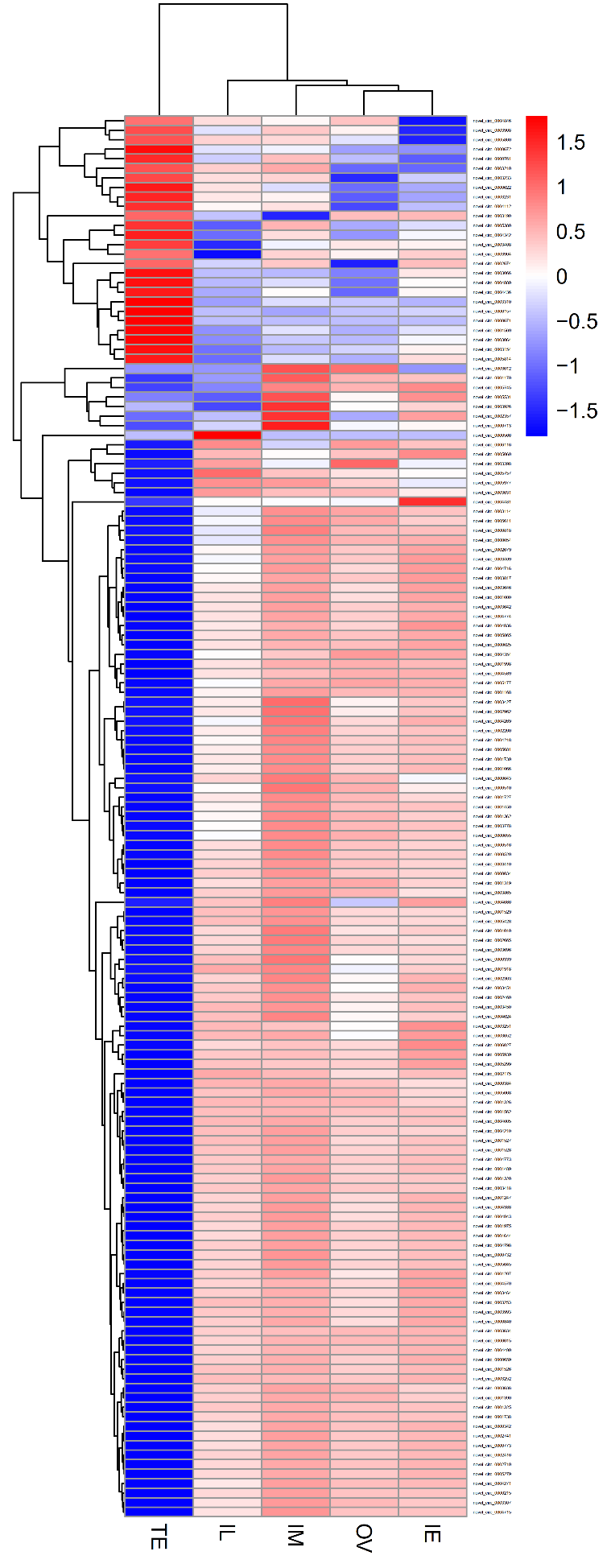


**Fig. S1 Expression patterns of differentially expressed circRNAs.** OV: ovary, IE: early intersexual gonad, IM: middle intersexual gonad, IL: late intersexual gonad, TE: testis.
