# Supplementary material for: Circular RNA expression profiles and CircSnd1-miR-135b/c-foxl2 axis analysis in gonadal differentiation of protogynous hermaphroditic ricefield eel Monopterus albus
Source: BMC Genomics. 2022 Aug 3;23:552. doi: 10.1186/s12864-022-08783-3 (PMC9347082; doi:10.1186/s12864-022-08783-3)
Supplement: Supplementary file 4 — Additional file 4. [file 12864_2022_8783_MOESM4_ESM.docx]

**Table S2 Differentially expressed circRNAs during the whole natural sex reversal**

| circRNAs ID |  | OV vs TE | IE vs TE | IM vs TE | IL vs TE |
| --- | --- | --- | --- | --- | --- |
| novel_circ_0003233 | FC | -11.685 | -7.9913 | -5.8447 | -4.1527 |
|  | pval | 1.78E-10 | 1.79E-10 | 5.34E-10 | 3.72E-04 |
|  | padj | 5.84E-08 | 6.40E-08 | 2.41E-07 | 7.84E-03 |
| novel_circ_0003231 | FC | -10.264 | -7.705 | -6.377 | -4.1271 |
|  | pval | 3.56E-10 | 5.85E-09 | 5.34E-10 | 1.57E-05 |
|  | padj | 5.85E-08 | 1.04E-06 | 2.41E-07 | 1.20E-03 |
| novel_circ_0004112 | FC | -8.5874 | -5.1985 | -4.5474 | -3.192 |
|  | pval | 7.27E-08 | 1.32E-04 | 1.27E-07 | 1.23E-05 |
|  | padj | 7.95E-06 | 3.91E-03 | 1.62E-05 | 1.20E-03 |
| novel_circ_0000672 | FC | -4.7009 | -5.2676 | -4.651 | -2.9077 |
|  | pval | 1.41E-04 | 1.47E-07 | 3.87E-05 | 2.58E-03 |
|  | padj | 5.82E-03 | 1.75E-05 | 1.25E-03 | 2.40E-02 |
| novel_circ_0000342 | FC | 6.0179 | 5.7472 | 5.9078 | 5.7042 |
|  | pval | 4.91E-03 | 3.41E-03 | 1.57E-03 | 3.70E-03 |
|  | padj | 4.74E-02 | 2.97E-02 | 1.73E-02 | 2.79E-02 |
| novel_circ_0000659 | FC | 6.1275 | 6.2095 | 5.91 | 6.6116 |
|  | pval | 4.08E-03 | 1.05E-03 | 1.68E-03 | 6.63E-04 |
|  | padj | 4.32E-02 | 1.38E-02 | 1.73E-02 | 1.16E-02 |
| novel_circ_0001325 | FC | 6.9894 | 6.5605 | 6.9271 | 7.5196 |
|  | pval | 3.67E-03 | 3.28E-04 | 6.52E-05 | 2.25E-05 |
|  | padj | 4.01E-02 | 6.55E-03 | 1.84E-03 | 1.28E-03 |
| novel_circ_0001526 | FC | 6.3625 | 6.4272 | 6.0973 | 7.1997 |
|  | pval | 3.22E-03 | 4.98E-04 | 7.88E-04 | 5.26E-05 |
|  | padj | 3.78E-02 | 8.46E-03 | 1.15E-02 | 2.34E-03 |
| novel_circ_0001527 | FC | 6.7942 | 6.73 | 7.4503 | 8.0041 |
|  | pval | 9.80E-04 | 2.03E-04 | 7.69E-06 | 3.62E-06 |
|  | padj | 2.30E-02 | 5.17E-03 | 4.34E-04 | 7.24E-04 |
| novel_circ_0001975 | FC | 7.2092 | 7.5241 | 7.9985 | 7.5913 |
|  | pval | 2.72E-04 | 2.20E-05 | 8.57E-07 | 1.81E-05 |
|  | padj | 9.92E-03 | 1.12E-03 | 7.73E-05 | 1.20E-03 |
| novel_circ_0002416 | FC | 6.5663 | 6.7041 | 6.5943 | 6.8079 |
|  | pval | 1.36E-03 | 2.46E-04 | 2.12E-04 | 1.69E-04 |
|  | padj | 2.97E-02 | 5.48E-03 | 4.34E-03 | 4.50E-03 |
| novel_circ_0002741 | FC | 7.3142 | 7.4138 | 7.0038 | 7.0884 |
|  | pval | 5.40E-04 | 2.96E-05 | 3.15E-03 | 1.14E-04 |
|  | padj | 1.77E-02 | 1.32E-03 | 2.68E-02 | 3.63E-03 |
| novel_circ_0003251 | FC | 5.0734 | 5.9778 | 4.6536 | 6.7107 |
|  | pval | 2.37E-03 | 3.32E-06 | 1.91E-04 | 6.89E-06 |
|  | padj | 3.78E-02 | 2.34E-04 | 4.10E-03 | 9.18E-04 |
| novel_circ_0003252 | FC | 6.6197 | 6.7554 | 6.3355 | 7.6913 |
|  | pval | 2.26E-03 | 5.66E-04 | 9.46E-03 | 1.83E-04 |
|  | padj | 3.78E-02 | 9.18E-03 | 4.74E-02 | 4.58E-03 |
| novel_circ_0003307 | FC | 6.4629 | 5.7452 | 6.4372 | 5.8486 |
|  | pval | 1.80E-03 | 4.45E-03 | 2.74E-04 | 3.30E-03 |
|  | padj | 3.69E-02 | 3.24E-02 | 5.15E-03 | 2.62E-02 |
| novel_circ_0003409 | FC | 5.0957 | 4.686 | 4.5516 | 5.028 |
|  | pval | 7.97E-04 | 6.05E-04 | 9.30E-05 | 7.48E-04 |
|  | padj | 2.18E-02 | 9.39E-03 | 2.33E-03 | 1.16E-02 |
| novel_circ_0003427 | FC | 4.1972 | 3.9376 | 4.6207 | 4.8401 |
|  | pval | 7.11E-04 | 3.92E-05 | 3.71E-09 | 2.21E-07 |
|  | padj | 2.12E-02 | 1.44E-03 | 8.36E-07 | 8.84E-05 |
| novel_circ_0003646 | FC | 4.9365 | 5.255 | 4.5448 | 5.3974 |
|  | pval | 2.84E-03 | 2.17E-04 | 1.06E-03 | 1.18E-04 |
|  | padj | 3.78E-02 | 5.17E-03 | 1.45E-02 | 3.63E-03 |
| novel_circ_0004269 | FC | 5.0531 | 5.0767 | 5.4685 | 5.0533 |
|  | pval | 2.30E-03 | 4.09E-04 | 2.04E-05 | 1.13E-03 |
|  | padj | 3.78E-02 | 7.38E-03 | 7.66E-04 | 1.46E-02 |
| novel_circ_0004271 | FC | 7.4505 | 7.3291 | 7.698 | 6.7198 |
|  | pval | 1.42E-04 | 4.04E-05 | 1.77E-05 | 2.49E-04 |
|  | padj | 5.82E-03 | 1.44E-03 | 7.25E-04 | 5.86E-03 |
| novel_circ_0004773 | FC | 6.7104 | 6.5623 | 6.8753 | 7.402 |
|  | pval | 9.42E-04 | 3.30E-04 | 6.08E-05 | 2.56E-05 |
|  | padj | 2.30E-02 | 6.55E-03 | 1.83E-03 | 1.28E-03 |
| novel_circ_0005865 | FC | 7.5885 | 8.0509 | 7.3921 | 7.0015 |
|  | pval | 1.38E-04 | 3.93E-06 | 1.39E-05 | 9.92E-05 |
|  | padj | 5.82E-03 | 2.34E-04 | 6.97E-04 | 3.63E-03 |

*Note:* FC refer to Log2 Fold Change. OV: ovary, IE: early intersexual gonad, IM: middle intersexual gonad, IL: late intersexual gonad, TE: testis.
