# Supplementary material for: Circular RNA expression profiles and CircSnd1-miR-135b/c-foxl2 axis analysis in gonadal differentiation of protogynous hermaphroditic ricefield eel Monopterus albus
Source: BMC Genomics. 2022 Aug 3;23:552. doi: 10.1186/s12864-022-08783-3 (PMC9347082; doi:10.1186/s12864-022-08783-3)
Supplement: Supplementary file 5 — Additional file 5. [file 12864_2022_8783_MOESM5_ESM.docx]

**Fig. S2** **GO enrichment of parent genes of DE circRNAs during the sexual reversal of ricefield eel.** OV: ovary, IM: middle intersexual gonad, IL: late intersexual gonad, TE: testis.
