# Supplementary material for: Circular RNA expression profiles and CircSnd1-miR-135b/c-foxl2 axis analysis in gonadal differentiation of protogynous hermaphroditic ricefield eel Monopterus albus
Source: BMC Genomics. 2022 Aug 3;23:552. doi: 10.1186/s12864-022-08783-3 (PMC9347082; doi:10.1186/s12864-022-08783-3)
Supplement: Supplementary file 6 — Additional file 6. [file 12864_2022_8783_MOESM6_ESM.docx]

**Table S3 Special circRNAs and involved KEGG pathways during sexual reversal**

| circRNAs ID | Parent gene | Pathway | group |
| --- | --- | --- | --- |
| novel_circ_0005865 | *spire2* | Dorso-ventral axis formation | OV/IE/IM/IL vs TE |
| novel_circ_0003251  novel_circ_0003252 | *pc* | Pyruvate metabolism  Citrate cycle (TCA cycle)  Carbon metabolism  Biosynthesis of amino acids  Metabolic pathways | OV/IE/IM/IL vs TE |
| novel_circ_0003253 |  |  |  |
| novel_circ_0004715  novel_circ_0004716 | *sdr16c5* | Retinol metabolism | OV/IE/IL vs TE |
| novel_circ_0005681 | *mthfr* | One carbon pool by folate  Carbon metabolism  Metabolic pathways | OV/IE/IM vs TE |
| novel_circ_0003114 | *pik3r3* | Progesterone-mediated oocyte maturation  VEGF signaling pathway  Toll-like receptor signaling pathway  ErbB signaling pathway  Adrenergic signaling in cardiomyocytes  mTOR signaling pathway  FoxO signaling pathway  Apoptosis  Jak-STAT signaling pathway  Regulation of actin cytoskeleton  Focal adhesion  Insulin signaling pathway  Phosphatidylinositol signaling system | OV /IM vs TE |
| novel_circ_0000761 | *gab1* | ErbB signaling pathway | IE vs TE |
| novel_circ_0005939 | *LOC109952263* | Progesterone-mediated oocyte maturation  Adrenergic signaling in cardiomyocytes  GnRH signaling pathway  Melanogenesis  Oocyte meiosis  Gap junction  Vascular smooth muscle contraction  Purine metabolism  Calcium signaling pathway | IE vs TE |
| novel_circ_0004889 | *aldh2* | Pyruvate metabolism  Lysine degradation  Glycerolipid metabolism  Histidine metabolism  beta-Alanine metabolism  Ascorbate and aldarate metabolism  Tryptophan metabolism  Valine, leucine and isoleucine degradation  Fatty acid degradation  Pentose and glucuronate interconversions  Arginine and proline metabolism  Glycolysis / Gluconeogenesis  Metabolic pathways | IM vs TE |
| novel_circ_0004005 | *akt1* | Progesterone-mediated oocyte maturation  VEGF signaling pathway  Toll-like receptor signaling pathway  ErbB signaling pathway  Tight junction  Adrenergic signaling in cardiomyocytes  mTOR signaling pathway  FoxO signaling pathway  Apoptosis  Jak-STAT signaling pathway  Focal adhesion  Insulin signaling pathway  Adipocytokine signaling pathway  MAPK signaling pathway | IL vs TE |
| novel_circ_0000034 | *atg7* | Regulation of autophagy | IL vs TE |

*Note:* OV: ovary, IE: early intersexual gonad, IM: middle intersexual gonad, IL: late intersexual gonad, TE: testis.
