# Supplementary material for: Circular RNA expression profiles and CircSnd1-miR-135b/c-foxl2 axis analysis in gonadal differentiation of protogynous hermaphroditic ricefield eel Monopterus albus
Source: BMC Genomics. 2022 Aug 3;23:552. doi: 10.1186/s12864-022-08783-3 (PMC9347082; doi:10.1186/s12864-022-08783-3)
Supplement: Supplementary file 7 — Additional file 7. [file 12864_2022_8783_MOESM7_ESM.docx]

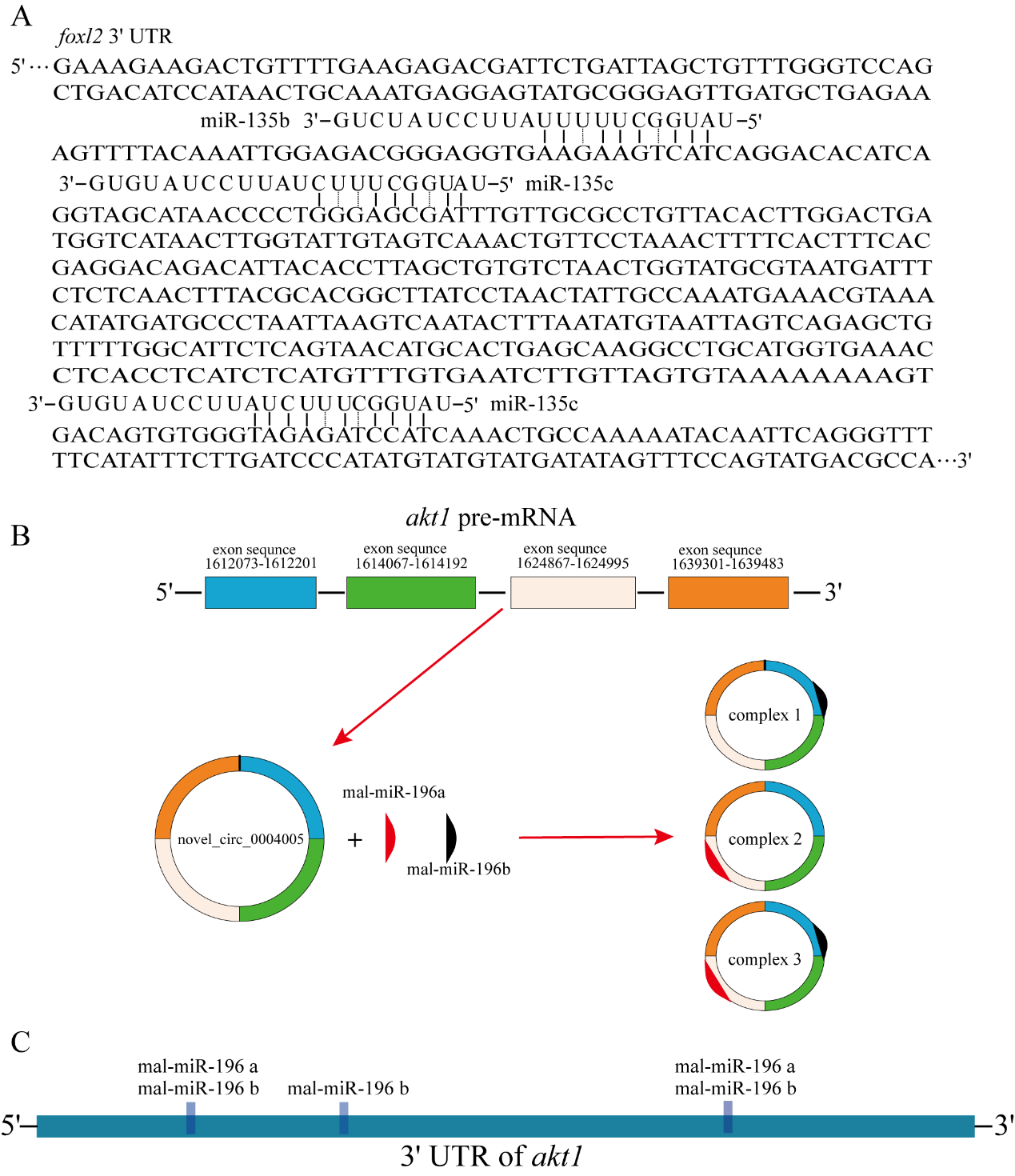


**Fig. S3 mal-miR-135b/c and mal-miR-196a/b binding sites were detected in 3' UTR of *foxl2*** (A) **and *akt1*** (C) **respectively.** The genomic loci of novel_circ_0004005, and mal-miR-196a/b may bind to novel_circ_0004005 to form miRNA-circRNA complex (B).
