# Supplementary material for: Circular RNA expression profiles and CircSnd1-miR-135b/c-foxl2 axis analysis in gonadal differentiation of protogynous hermaphroditic ricefield eel Monopterus albus
Source: BMC Genomics. 2022 Aug 3;23:552. doi: 10.1186/s12864-022-08783-3 (PMC9347082; doi:10.1186/s12864-022-08783-3)
Supplement: Supplementary file 8 — Additional file 8. [file 12864_2022_8783_MOESM8_ESM.docx]

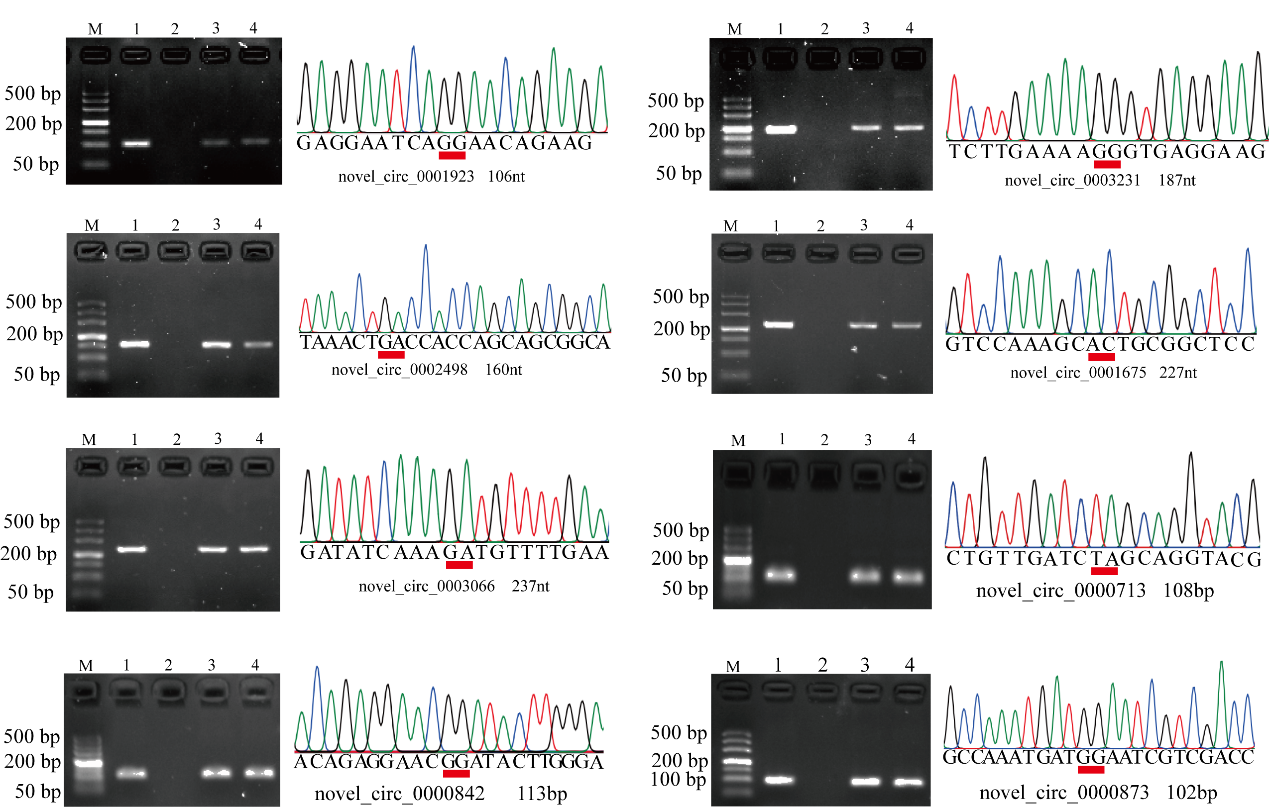


**Fig. S4 Characterization of predicted circRNAs.** Amplification of circRNAs using divergent primers with cDNA. 1, 3 and 4 lanes refer to cDNA samples transcribed by Random hexamer primer, 2 lanes refer to cDNA samples transcribed by Oligo(dT)_18_. The cDNA used in lane 3 was synthesized by total RNA that digested with RNase R, and lane 4 was control.
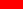
: Represents back-splicing sties.
