# Supplementary material for: Circular RNA expression profiles and CircSnd1-miR-135b/c-foxl2 axis analysis in gonadal differentiation of protogynous hermaphroditic ricefield eel Monopterus albus
Source: BMC Genomics. 2022 Aug 3;23:552. doi: 10.1186/s12864-022-08783-3 (PMC9347082; doi:10.1186/s12864-022-08783-3)
Supplement: Supplementary file 9 — Additional file 9. [file 12864_2022_8783_MOESM9_ESM.docx]

**Table S4 Sequence of primers for circRNAs validation**

| Gene | Primer sequences | Tm/℃ | Length/bp |
| --- | --- | --- | --- |
| *ef1α* | F: CGCTGCTGTTTCCTTCGTCC | 55.3 | 102 |
|  | R: TTGCGTTCAATCTTCCATCCC |  |  |
| *rpl17* | F: GACTAAATCATGCAAGTCGAGGG | 56.2 | 160 |
|  | R: GTTGTAGCGACGGAAAGGGAC |  |  |
| novel_circ_0000713 | F: CTCGTACCTGCTAGATCAACA | 54.3 | 108 |
|  | R: CGATGGGTCCAATAGGCTCA |  |  |
| novel_circ_0000842 | F: CTGCCCAGTAACTACGACAA | 57.6 | 113 |
|  | R: CCCAAGTATCCGTTCCTCT |  |  |
| novel_circ_0001923 | F: GGAGAGGGAGGAATCAGGAA | 56.0 | 106 |
|  | R: GCTTTGCGTCTAGGGTTGTT |  |  |
| novel_circ_0002498 | F: CCCAAGTCCAATAAACTGACCA | 58.1 | 160 |
|  | R: ATTTTCCAGAAGCAAAGAGCG |  |  |
| novel_circ_0001569 | F: GACAGCGGAATCATAAGAAGAA | 53.9 | 341 |
|  | R: GGAGTTACATCAGTGCCCTTTGC |  |  |
| novel_circ_0003066 | F: CGAGGGTGGAGGCTCAGACAGT | 56.5 | 237 |
|  | R: TGAGAACGGAGGCAGCAGCAGT |  |  |
| novel_circ_0003409 | F: GACACTGGTCAACTAATTGTAT | 57.2 | 186 |
|  | R: ATCTGTGAAGGTCTGTCTCTAAAG |  |  |
| novel_circ_0003231 | F: AGATGTGAACAGATGAGGAAAC | 55.3 | 187 |
|  | R: ATTTCTCCTGTCCTCCATACTC |  |  |
| novel_circ_0001675 | F: TGGAGGATTCTGGGAATTACAC | 58.2 | 227 |
|  | R: CTTCAGTTCGTTGCCATCTTTG |  |  |
| novel_circ_0000873 | F: ACGTGGTTTTGGACACATTG | 56.3 | 102 |
|  | R: CCATCATTTGGCTTCTTGAC |  |  |
