# Supplementary material for: Circular RNA expression profiles and CircSnd1-miR-135b/c-foxl2 axis analysis in gonadal differentiation of protogynous hermaphroditic ricefield eel Monopterus albus
Source: BMC Genomics. 2022 Aug 3;23:552. doi: 10.1186/s12864-022-08783-3 (PMC9347082; doi:10.1186/s12864-022-08783-3)
Supplement: Supplementary file 10 — Additional file 10. [file 12864_2022_8783_MOESM10_ESM.docx]

**Table S5 Sequence of primers for RNA immunoprecipitation**

| Gene | Primer sequences | Tm/℃ | Length/bp |
| --- | --- | --- | --- |
| *foxl2* | F: GTTGGCAGAACAGCATCAGA | 56.5 | 135 |
|  | R: TCCCCTTCTCAAACATGTCC |  |  |
| mal-miR-135c | F: GGGTATGGCTTTCTATTCC | 63.8 | - |
|  | R: CAGTGCGTGTCGTGGAGT |  |  |
| mal-miR-135b | F: GGGTATGGCTTTTTATTCC | 64.2 | - |
|  | R: CAGTGCGTGTCGTGGAGT |  |  |
| *circSnd1* | F: AGCTCATTGGTAAAAAGCCA | 55.8 | 124 |
|  | R: CTTGAATGTGGGACACTTGA |  |  |
| *snd1* | F: GGAAAGACACAACAGGCGA | 56.3 | 295 |
|  | R: AGCAAGGCACGAACAACAC |  |  |
