# Supplementary material for: Circular RNA expression profiles and CircSnd1-miR-135b/c-foxl2 axis analysis in gonadal differentiation of protogynous hermaphroditic ricefield eel Monopterus albus
Source: BMC Genomics. 2022 Aug 3;23:552. doi: 10.1186/s12864-022-08783-3 (PMC9347082; doi:10.1186/s12864-022-08783-3)
Supplement: Supplementary file 11 — Additional file 11. [file 12864_2022_8783_MOESM11_ESM.docx]

**Table S6 Sequence of primers for *circSnd1* separation of cytoplasm and nucleus**

| Gene | Primer sequences | Tm/℃ | Length/bp |
| --- | --- | --- | --- |
| *ef1α* | F: CGCTGCTGTTTCCTTCGTCC | 55.3 | 102 |
|  | R: TTGCGTTCAATCTTCCATCCC |  |  |
| *rpl17* | F: GACTAAATCATGCAAGTCGAGGG | 56.2 | 160 |
|  | R: GTTGTAGCGACGGAAAGGGAC |  |  |
| 18s | F: ACGAACAAGACTCCAGCAT | 54.1 | 129 |
|  | R: ACATCTAAGGGCATCACAG |  |  |
| U6 | F: GGGTTACTTTGGTAGCACAT | 55.2 | 100 |
|  | R: GAGGAACGCTTCACGAATTT |  |  |
| *circSnd1* | F: TCAACGCTGATGCTTTGGTGGT | 56.5 | 341 |
|  | R: CTGCGAATGGCTCTGGTGTCTC |  |  |
